# Understanding the Link Between Toxic Leadership and Employee Well-being: The Interplay of Employee Turnover, Organizational commitment, Shift timing & Industry Sector

**DOI:** 10.12688/f1000research.171577.1

**Published:** 2026-01-09

**Authors:** Abhishek Shukla, BR Santosh, Sangeetha Vinod

**Affiliations:** 1 Manipal Academy of Higher Education - Dubai Campus, Academic City, Dubai, United Arab Emirates; 2 Manipal Academy of Higher Education, Manipal, Karnataka, India; 3 Manipal Academy of Higher Education - Dubai Campus, Academic City, Dubai, United Arab Emirates

**Keywords:** Toxic Leadership, Employee Well-being, Employee Turnover, Organizational Commitment, Shift Timing, Industry Sector, Quantitative Research, UAE Workforce

## Abstract

**Background:**

This study examines the impact of toxic leadership on employee well-being and turnover across various industry sectors in the United Arab Emirates. Toxic leadership, marked by manipulative and hostile behaviors, has been associated with negative employee outcomes. However, limited research has jointly explored these effects with the moderating roles of organizational commitment and shift timing in this regional context. Grounded in Social Exchange Theory, Psychological Contract Theory, and Conservation of Resources Theory, the study investigates how toxic leadership influences employee well-being and turnover and whether organizational commitment, shift timing, and industry sector moderate these relationships.

**Methods:**

A quantitative survey design was used, involving responses from 300 employees representing diverse sectors. Regression analysis and structural equation modeling were applied to assess the direct and moderating effects of toxic leadership on well-being and turnover. The analysis examined model significance, variance explained, and moderation through interaction terms while confirming validity and reliability through AVE, HTMT, and VIF metrics.

**Results:**

Toxic leadership significantly reduced employee well-being (R = 0.445, R ^2^ = 0.198) and increased turnover (R = 0.422, R ^2^ = 0.178). Organizational commitment moderated the toxic leadership–turnover relationship (B = 0.2124, p = .0088) but did not moderate the toxic leadership–well-being relationship. Shift timing and industry sector showed minimal moderation with negligible variance explained. The structural model demonstrated good fit and explanatory power for turnover (R ^2^ = 0.582) and workplace behavior (R ^2^ = 0.373), confirming acceptable reliability and validity indices.

**Conclusion:**

Toxic leadership detrimentally affects employee well-being and retention across all sectors. Organizational commitment partially buffers these effects on turnover but not on well-being. Findings highlight that reducing toxic leadership behaviors is essential for employee health and organizational stability, as contextual factors like shift timing or sector offer limited protective influence.

## 1. Introduction

In today’s competitive and ever-changing organizational environment, leadership plays a vital role in shaping employee experiences and outcomes. While positive leadership styles promote motivation, satisfaction, and performance, the negative effects of toxic leadership have gained increasing attention. Toxic leadership involves behaviors such as manipulation, hostility, and authoritarian control, which can harm both individual well-being and organizational effectiveness. As noted by Tanuwijaya and Jakaria (2022), toxic leadership fosters a hostile work climate that undermines psychological safety and morale. Iqbal et al. (2022) also highlight that such leadership increases stress and burnout, leading to a decline in overall employee well-being. These outcomes can be understood through Conservation of Resources Theory, which suggests that toxic environments drain employees’ emotional and psychological resources.

The impact of toxic leadership extends to increased employee turnover, as employees often choose to leave in search of healthier workplaces. According to Ofei et al. (2023), employees unable to manage the pressures of toxic leadership often resign. This aligns with Social Exchange Theory and Psychological Contract Theory, where broken expectations and imbalanced relationships drive employee exit. However, the effects of toxic leadership may vary. Nonehkaran et al. (2023) propose that organizational commitment can serve as a buffer, while Reyhanoglu and Akin (2022) identify shift timing as a possible moderator. This study investigates these dynamics to support better human resource strategies and leadership development.

## 2. Research aim and objectives

The study aims to understand the link between Toxic Leadership, Employee Well-being, Employee Turnover, Organizational commitment, Shift timing and Industry Sector.

The research addresses the following research objectives:

1. To assess the impact of Toxic Leadership on the Well-being of the employees.
2. To understand the impact of Toxic Leadership on Employee Turnover.
3. To examine the moderating roles of organizational commitment and shift timing between Toxic Leadership and Employee Well-being.
4. To examine the moderating roles of organizational commitment and shift timing between Toxic Leadership and Employee Turnover.

## 3. Literature review

### 3.1 Introduction

The researcher surveyed the abstracted academic journals, conference proceedings, technical reports, books, and other relevant publications to analyse and review the secondary literature sources. It emphasises the following subsections.

### 3.2 Toxic leadership and employee well-being

In recent years, academic interest in toxic leadership has increased notably, reflecting its rising importance in modern organizational settings. Waldiya (2023) explored how toxic leadership affects employee well-being and found no significant relationship with personal well-being. However, the study identified a strong association between toxic leadership and emotional exhaustion. It also uncovered a meaningful link between toxic leadership behaviors and perceived roughness in managerial conduct. These findings support Conservation of Resources Theory, which suggests that toxic leaders deplete employees’ emotional resources, leading to exhaustion and strained perceptions of authority.

Expanding on the psychological processes involved, Hadadian and Sayadpour (2018) investigated workplace anxiety as a mediating factor between toxic leadership and employee well-being. Their research demonstrated both direct and indirect effects of toxic leadership, organizational constraints, interpersonal conflicts, and workload on employment-related well-being. These variables jointly explained thirteen percent of the variance in well-being. Furthermore, toxic leadership alone accounted for twelve percent of the variation in interpersonal conflict. This aligns with Social Exchange Theory, which emphasizes the breakdown of trust and fairness under toxic leadership, further eroding workplace relationships.

In a different setting, Naeem and Khurram (2020) studied toxic leadership in Pakistan’s banking sector and confirmed its negative impact on employee well-being, staff engagement, and turnover intentions. Employee well-being and engagement acted as partial mediators in the link between toxic leadership and turnover, illustrating how psychological contracts are violated in toxic work environments.

In the healthcare field, Koç et al. (2022) examined the effects of toxic leadership on retaliatory behavior among nurses. The study found a significant link between toxic leadership and vindictive actions, although psychological health did not moderate this relationship. This reflects how continued exposure to toxic leadership fosters revenge-seeking behavior, highlighting the psychological toll of such environments, especially in emotionally demanding roles.

### 3.3 Toxic leadership and employee turnover

Soomro et al. (2024) examined the presence of toxic chief executive officers in microfinance institutions and explored their influence on employee engagement, psychological well-being, and resignation intentions. Their findings revealed that toxic leadership significantly impacts mental health and employee involvement. A partial mediation was identified among toxic leadership, well-being, staff engagement, and turnover intention. These findings align with Conservation of Resources Theory, as toxic leaders can deplete essential emotional and psychological resources, reducing employee resilience and motivation. The results also support Social Exchange Theory by illustrating how imbalanced and harmful interactions between leaders and employees reduce reciprocal commitment and increase resignation intentions. The study highlights the need for institutions to foster healthy and supportive environments to retain and engage staff.

In the healthcare sector, Bakkal et al. (2019) developed a conceptual model investigating how toxic leadership affects job satisfaction and turnover intention, with job contentment as a mediator. Their study found strong links between narcissistic leadership and low job fulfillment. A statistically significant connection also emerged between perceived ungratefulness and employees’ desire to leave. Emotional distress, a key outcome of toxic leadership, further contributed to intentions to resign, supporting the Conservation of Resources framework.

Leadership style remains central to shaping employee behavior across sectors. Constructive leadership enhances organizational performance, while toxic leadership diminishes engagement and productivity. Hattab et al. (2022), drawing on Psychological Contract Theory, showed that when employees perceive a breach in expected treatment due to toxic leadership, they are more likely to develop turnover intentions and engage in counterproductive behaviors.

Nunes and Palma-Moreira (2024) investigated how burnout mediates the relationship between toxic leadership and resignation intention. Toxic leadership was found to increase both burnout and turnover intention. Among Portuguese workers, burnout partially mediated this relationship through disengagement, while a complete mediation was observed among Angolan employees. These results further support the Conservation of Resources and Psychological Contract frameworks by showing how toxic leadership undermines employees’ mental energy and workplace expectations, leading to higher attrition.

### 3.4 Organizational commitment, shift timings, employee well-being, and employee turnover

Suárez-Albanchez et al. (2021) examined the influence of job-related safety and health regulations on employees’ commitment to both their roles and their organizations, and how this ultimately affects their intentions to resign. The study established a negative relationship between workplace safety and health policies and the intention to leave, along with a positive relationship between those regulations and both job involvement and organizational commitment. In addition, the results revealed that employees’ job performance and organizational loyalty are negatively linked to their desire to exit, suggesting that improved regulatory frameworks enhance engagement and reduce resignation tendencies.

In the hospitality sector, Dorta-Afonso et al. (2021) explored the pathways through which high performance work systems affect employees’ satisfaction and psychological well-being. Their investigation confirmed that such systems directly improve job satisfaction, motivation, commitment to the organization, and overall quality of life. Furthermore, organizational commitment and motivation significantly influence job satisfaction, which in turn contributes positively to life quality. The study also found that individual job performance is greatly enhanced when employees report high levels of satisfaction and well-being, highlighting the essential role of supportive workplace systems in performance outcomes.

Focusing on the unique pressures during the COVID-19 crisis, Serhan et al. (2022) investigated how organizational commitment influences the turnover intentions of Islamic bank employees. The results revealed significant associations among emotional, normative, and continuance commitment, organizational loyalty, individual differences, and resignation intentions. Each dimension of organizational commitment was shown to reduce the desire to leave the job. Furthermore, the relationship between commitment and turnover intention was moderated by both employee-specific traits and perceived organizational performance, suggesting that retention strategies must be tailored to individual and contextual factors.

Suter et al. (2020) evaluated how employees in high-intensity mental health environments respond to a newly introduced 12-hour work shift from a well-being perspective. The findings indicated that such extended shift structures produce varied effects on worker well-being, with notably harmful consequences in high-stress and complex organizational contexts. The research emphasized the importance of worker autonomy and flexibility for maintaining well-being and staff retention, particularly in aging and competitive labor markets. Consequently, the study recommended that imposed 12-hour shift systems should be reconsidered or discontinued to preserve employee health.

Applying the Guidelines for Worker Well-Being, McHugh et al. (2020) investigated how manufacturing employees perceive the effects of shift work. Participants reported that shift work had a detrimental influence on well-being in four out of five framework categories. However, in the area of workplace culture and employment norms, some workers viewed shift schedules as necessary, fair, and financially beneficial. These mixed responses underscore that the impact of shift work is highly dependent on contextual and perceptual factors, reinforcing the need for nuanced organizational policies that account for both risks and benefits of such employment structures.

### 3.5 Research gap

Although studies have been independently conducted previously on the aspects of Toxic Leadership (TL), Employee Well-being (EW), Employee Turnover (ET), Organizational Commitment (OM), Shift Timings (ST), and Industry Sector, there exists a major research gap that fails to ascertain the link between all these variables. Also, no study as per the researcher’s knowledge, has been conducted previously that aims to establish the link between all these variables in the context of firms belonging to different industries in the UAE. The current study, therefore, aims to address this research gap and add crucial insights pertaining to the domain of toxic leadership and its impact on employee well-being and employee turnover.

### 3.6 Hypothesis development

Based on the review of the literature, the following hypothesis has been proposed.

Ha1: Toxic Leadership has a significant impact on the well-being of employees.

H01: Toxic Leadership does not have a significant impact on the well-being of employees.

Ha2: Toxic Leadership has a significant impact on Employee Turnover.

H02: Toxic Leadership does not have a significant impact on Employee Turnover.

Ha3: Organizational Commitment plays a moderating role between Toxic Leadership and Employee Well-being.

H03: Organizational Commitment does not play a moderating role between Toxic Leadership and Employee Well-being.

Ha4: Shift timings play a moderating role between Toxic Leadership and Employee Well-being.

H04: Shift timings do not play a moderating role between Toxic Leadership and Employee Well-being.

Ha5: Organizational commitment plays a moderating role between Toxic Leadership and Employee Turnover.

H05: Organizational commitment does not play a moderating role between Toxic Leadership and Employee Turnover.

Ha6: Shift timings play a moderating role between Toxic Leadership and Employee Turnover.

H06: Shift timings do not play a moderating role between Toxic Leadership and Employee Turnover.

Ha7: Toxic Leadership’s impact on Employee Outcomes varies in the context of different industries.

H07: Toxic Leadership’s impact on Employee Outcomes does not vary in the context of different industries.

### 3.7 Conceptual framework

**Figure 1.**
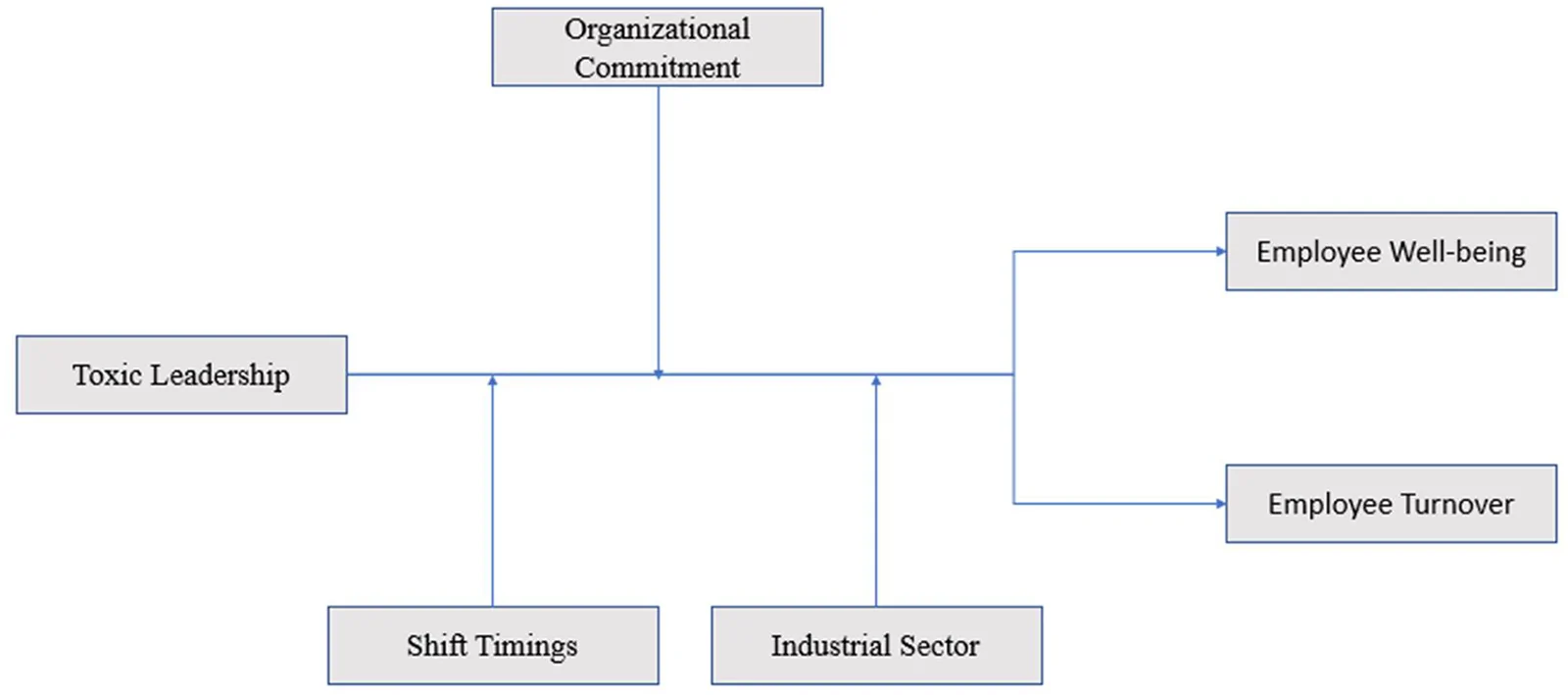
Conceptual framework showing the relationship between the dependent, independent, moderator and mediator variables.

The relationships among variables are illustrated in the conceptual model in Figure 1.

## 4. Research methodology

This study adopts a positivist research paradigm, which emphasizes objectivity, measurement, and hypothesis testing, allowing for empirical assessment of relationships among variables ( Junjie & Yingxin, 2022; Sanchez et al., 2023). A quantitative research approach was chosen to ensure precision and generalizability through numerical data analysis ( Jamieson et al., 2023; Ghanad, 2023). The research follows a descriptive research design, which is suitable for systematically describing the nature and strength of relationships among toxic leadership, employee well-being, turnover, organizational commitment, shift timing, and industry sector ( Taherdoost, 2022; Furidha, 2023). Data was collected using a structured Likert-scale survey, which is widely used for measuring attitudes and perceptions in organizational settings ( Jebb et al., 2021; South et al., 2022). The target population includes all employees working across various sectors in the UAE, ensuring a broad and inclusive understanding of workplace dynamics. The overall methodology of the research process is outlined in Table 1.

**Table 1.** RM flow chart.

	Initial
My supervisor/leader demeans or belittles the team frequently.	1.000
Supervisors in our organisation take credit for work done by subordinates.	1.000
Our supervisor shows favouritism toward specific employees, creating a toxic work environment.	1.000
Our supervisor/leader blames others for their own mistakes or failures.	1.000
Our supervisor is more concerned with their personal gain compared to team success or employee well-being.	1.000
I feel emotionally and mentally healthy at my workplace.	1.000
I feel enthusiastic and motivated to perform my day-to-day tasks.	1.000
I have a good work-life balance that allows me to rest and give time to my family.	1.000
I feel that my work contributes to my personal growth and fulfillment.	1.000
I can openly communicate my concerns or challenges at work.	1.000
I frequently think about leaving my current job.	1.000
If I received a better offer, I would leave this company immediately.	1.000
The lack of career growth opportunities often motivates me to consider leaving.	1.000
Poor leadership in my department has made me consider quitting.	1.000
I feel my contributions are not recognised, which affects my desire to stay in the organisation.	1.000
I feel a strong sense of belonging to my company.	1.000
I feel emotionally attached to this company.	1.000
It would be very hard for me to leave this organisation right now, even if I wanted to.	1.000
In my opinion, loyalty to one’s organisation is important and should be maintained.	1.000
I am committed to the success and goals of this company.	1.000
My shift timing lets me maintain a healthy work-life balance.	1.000
My existing shift does not interfere with my social or family life.	1.000
My job satisfaction is affected by the timing of my shift.	1.000
I feel well-rested and alert throughout my shift.	1.000
I am satisfied with my existing shift timing.	1.000

Extraction Method: Principal Component Analysis.

Data was collected between November 2023 and January 2024. A sample of 300 employees was selected using stratified random sampling, with strata based on industry size, sector, and shift timing, to enhance representativeness and control for contextual differences ( Hossan et al., 2023; Rahman et al., 2022). This methodological framework ensures that the study’s findings are reliable, valid, and applicable across multiple industry contexts.

**Ethical considerations**

This study did not require prior ethical approval, as it involved an anonymous, non-interventional survey with no collection of personal or sensitive data. Participants were fully informed about the purpose of the study and responded voluntarily. The data collection process strictly adhered to academic research standards.

**Informed consent**

All participants provided verbal informed consent prior to completing the survey. Participants were comfortable for the survey but were particular on verbal consent due to cultural sensitivity, anonymity concerns, and the non-intrusive nature of the study. Hence, this was opted for the study. Participation was voluntary, and responses were anonymous. No personal identifiers were collected.

### 4.1 Statistical analysis

Data analysis was conducted using SPSS, applying statistical techniques such as correlation, regression, ANOVA, and structural equation modeling (SEM) to examine relationships among variables and test hypotheses. These methods are appropriate for identifying both direct and moderating effects in quantitative research ( Attwal & Singh, 2024; Okagbue et al., 2021). Correlation and regression helped assess associations and predictive strength, while ANOVA explored group differences based on shift timing and industry sector. SEM enabled comprehensive modeling of complex relationships ( Watkins, 2021; Purwanto & Sudargini, 2021), ensuring a robust analysis of toxic leadership’s impact on employee outcomes.

Table 2 presents the communalities analysis showing total variance explained across variables. The communalities analysis using Principal Component Analysis reveals that all items initially have communalities of 1.000, indicating that each variable’s total variance is considered in the extraction process. However, since only initial communalities are provided and no extracted values are shown, it is not possible to assess how much variance in each item is explained by the underlying components after extraction. Initial communalities of 1.000 are typical before factor extraction, as they represent the assumption that each item’s variance is fully accounted for. To interpret the effectiveness of the factor solution, the extracted communalities would be required to evaluate shared variance among the variables.

**Table 2.** Communalities analysis.

Total Variance Explained						
Component	Initial Eigenvalues			Rotation Sums of Squared Loadings		
	Total	% of Variance	Cumulative %	Total	% of Variance	Cumulative %
1	8.531	34.126	34.126	5.684	22.735	22.735
2	4.722	18.889	53.015	4.863	19.45	42.186
3	3.724	14.895	67.91	3.506	14.025	56.21
4	2.164	8.658	76.568	3.211	12.845	69.055
5	1.317	5.266	81.834	2.463	9.851	78.907
6	1.046	4.184	86.018	1.778	7.112	86.018
7	0.912	3.647	89.665			
8	0.755	3.021	92.687			
9	0.577	2.308	94.995			
10	0.347	1.387	96.381			
11	0.237	0.947	97.329			
12	0.199	0.794	98.123			
13	0.164	0.656	98.779			
14	0.11	0.441	99.22			
15	0.074	0.295	99.515			
16	0.066	0.265	99.78			
17	0.049	0.196	99.976			
18	0.006	0.024	100			
19	1.26E-16	5.03E-16	100			
20	6.80E-17	2.72E-16	100			
21	8.88E-18	3.55E-17	100			
22	-3.88E-33	-1.55E-32	100			
23	-2.30E-17	-9.18E-17	100			
24	-1.06E-16	-4.24E-16	100			
25	-2.20E-16	-8.81E-16	100			

As shown in Table 3, six components accounted for over 86% of the total variance. The communalities analysis indicates that all 25 items initially had communalities of 1.000, reflecting that each item’s total variance was included before factor extraction. Using Principal Component Analysis, six components were extracted with eigenvalues greater than 1. Together, these components explain 86.02 percent of the total variance. The rotation sums of squared loadings reveal that the first component explains 22.74 percent, the second 19.45 percent, and the third 14.03 percent of the variance, cumulatively reaching 56.21 percent. This suggests a strong factor structure with meaningful underlying dimensions. However, the actual communalities after extraction would be needed to assess how well each item is represented.

**Table 3.** TVA analysis.

Rotated Component Matrix						
	Component					
	1	2	3	4	5	6
My supervisor/leader demeans or belittles the team frequently.		0.969				
Supervisors in our organisation take credit for work done by subordinates.		0.877				
Our supervisor shows favouritism toward specific employees, creating a toxic work environment.	0.95					
Our supervisor/leader blames others for their own mistakes or failures.			0.958			
Our supervisor is more concerned with their personal gain compared to team success or employee well-being.			0.958			
I feel emotionally and mentally healthy at my workplace.	0.514					
I feel enthusiastic and motivated to perform my day-to-day tasks.	0.95					
I have a good work-life balance that allows me to rest and give time to my family.	0.765					
I feel that my work contributes to my personal growth and fulfillment.	0.829					
I can openly communicate my concerns or challenges at work.					0.879	
I frequently think about leaving my current job.					0.793	
If I received a better offer, I would leave this company immediately.				0.83		
The lack of career growth opportunities often motivates me to consider leaving.				0.691		
Poor leadership in my department has made me consider quitting.				0.815		
I feel my contributions are not recognised, which affects my desire to stay in the organisation.				0.839		
I feel a strong sense of belonging to my company.					0.667	0.531
I feel emotionally attached to this company.						0.77
It would be very hard for me to leave this organisation right now, even if I wanted to.						0.693
In my opinion, loyalty to one’s organisation is important and should be maintained.	0.66					
I am committed to the success and goals of this company.	0.56					
My shift timing lets me maintain a healthy work-life balance.		0.969				
My existing shift does not interfere with my social or family life.		0.877				
My job satisfaction is affected by the timing of my shift.	0.95					
I feel well-rested and alert throughout my shift.			0.958			
I am satisfied with my existing shift timing.		0.969				

Extraction Method: Principal Component Analysis.

Rotation Method: Varimax with Kaiser Normalization.

Table 4 displays the rotated component matrix with distinct item loadings across six components. The rotated component matrix from the Principal Component Analysis with Varimax rotation reveals a clear factor structure, with items strongly loading onto six distinct components. Items related to toxic leadership, employee well-being, turnover intentions, organizational commitment, and shift satisfaction load highly on separate components, indicating well-defined constructs. For instance, toxic leadership items load above 0.95 on Component 1, while turnover-related items load strongly on Component 3. Shift-related items also show high loadings on Component 6. This structure supports the validity of the questionnaire in capturing multiple dimensions. The strong loadings across items indicate that the extracted components effectively explain the shared variance among variables.

**Table 4.** Rotated component matrix analysis.

Model Summary				
Model	R	R Square	Adjusted R Square	Std. Error of the Estimate
1	.445 ^a^	0.198	0.195	0.48701

Anova						
Model		Sum of Squares	df	Mean Square	F	Sig.
1	Regression	17.424	1	17.424	73.461	.000 ^b^
	Residual	70.68	298	0.237		
	Total	88.103	299			

Coefficients						
Model		Unstandardized Coefficients		Standardized Coefficients	t	Sig.
		B	Std. Error	Beta		
1	(Constant)	1.659	0.272		6.096	0
	Toxic Leadership	0.519	0.061	0.445	8.571	0

^a^ Predictors: (Constant), Toxic Leadership.

^b^ Dependent Variable: Well-being of the employees.

## 5. Findings and discussion

As can be seen in Table 5, The regression analysis showed that toxic leadership significantly impacts employee well-being with R = 0.445 and R squared = 0.198 indicating it explains 19.8 percent of the variance. ANOVA confirmed the model’s significance with F value 73.46 and p less than 0.001. The coefficient B = 0.519 suggests a moderate positive effect. Based on Social Exchange Theory and Psychological Contract Theory this implies that negative leadership disrupts trust and perceived fairness. Conservation of Resources Theory supports that such stress depletes emotional and psychological resources.

**Table 5.** Hypothesis 1 analysis.

Model Summary				
Model	R	R Square	Adjusted R Square	Std. Error of the Estimate
1	.422 ^a^	0.178	0.175	0.44275

Anova						
Model		Sum of Squares	df	Mean Square	F	Sig.
1	Regression	12.647	1	12.647	64.518	.000 ^b^
	Residual	58.416	298	0.196		
	Total	71.063	299			

^a^ Predictors: (Constant), Toxic Leadership.

^b^ Dependent Variable: Employee Turnover.

The results in Table 6 confirm that toxic leadership significantly influences employee turnover. The results support Ha2, indicating that toxic leadership has a significant impact on employee turnover. The model is statistically significant (R = 0.422, R ^2^ = 0.178, F (1, 298) = 64.518, p < .001), explaining 17.8% of the variance in turnover. The regression coefficient for toxic leadership is also significant (B = 0.443, β = 0.422, t = 8.032, p < .001), showing a moderate positive effect. Therefore, H02 is rejected, confirming that increased toxic leadership is associated with higher employee turnover, emphasizing the need for corrective organizational strategies.

**Table 6.** Hypothesis 2 analysis.

Coefficients ^a^						
Model		Unstandardized Coefficients		Standardized Coefficients	t	Sig.
		B	Std. Error	Beta		
1	(Constant)	2.045	0.247		8.266	0
	Toxic Leadership	0.443	0.055	0.422	8.032	0

^a^ Dependent Variable: Employee Turnover.

**Figure 2.**
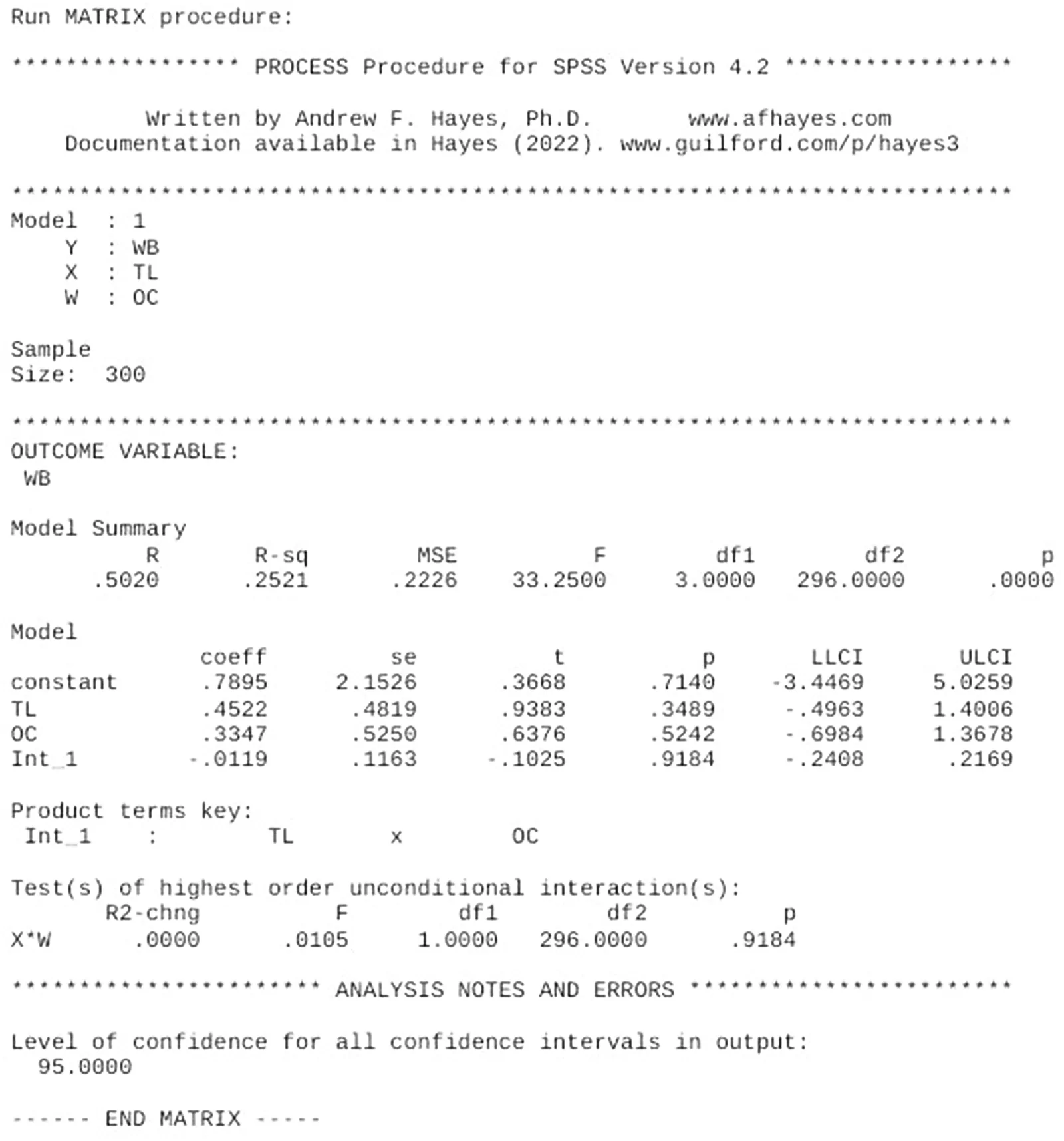
Regression output showing the moderating effect of organizational commitment on TL and EW.

**Figure 3.**
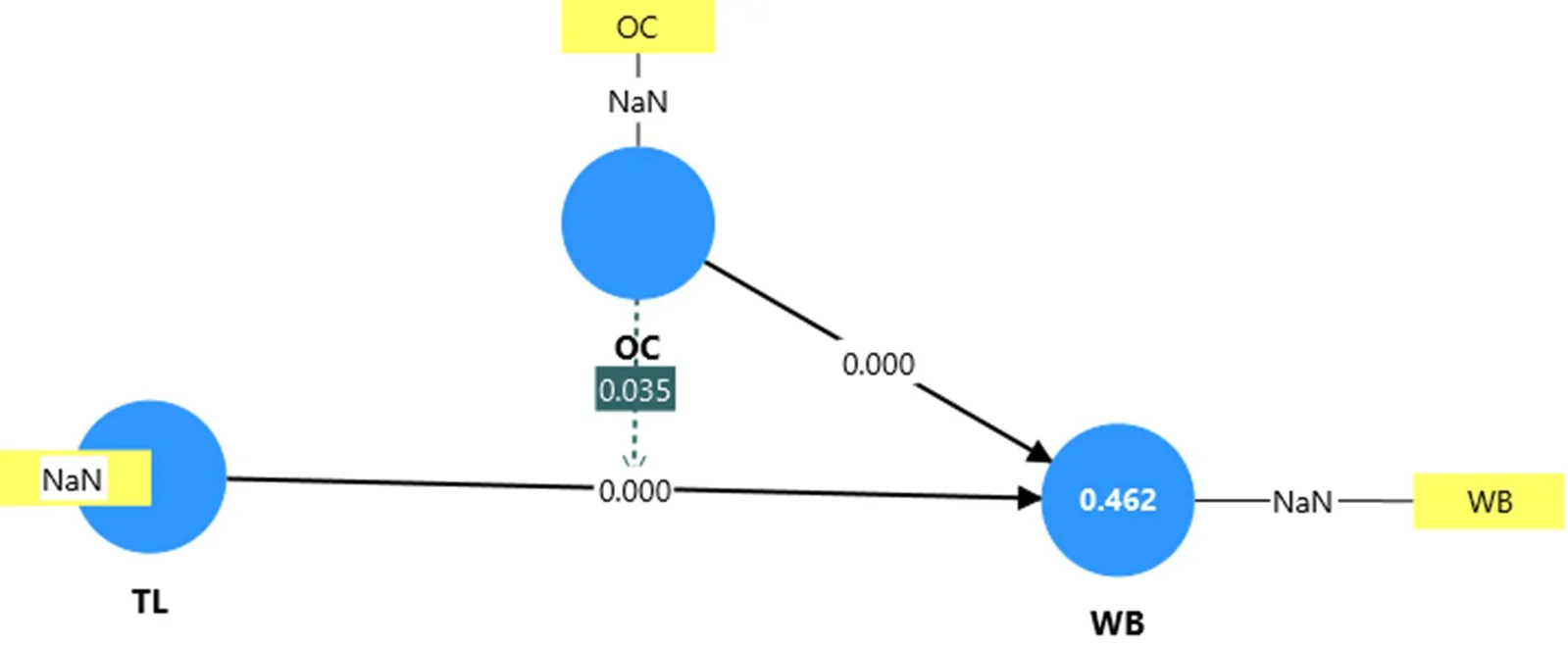
Interaction plot illustrating the non-significant moderating interaction effect of Organizational commitment.

The model significantly explains 25.2% of the variance in employee well-being (R ^2^ = 0.2521, F (3, 296) = 33.25, p < .001), but the interaction between toxic leadership and organizational commitment is not significant (B = –0.0119, p = .9184, 95% CI [–0.2408, 0.2169]; R ^2^ change = 0.0000). Despite one model indicating a significant p-value (0.00), the confidence interval includes zero, and the R ^2^ change is negligible, suggesting no meaningful moderation. These findings imply that organizational commitment does not reliably buffer the negative impact of toxic leadership on well-being. Therefore, interventions should focus on directly addressing toxic leadership, rather than relying on commitment as a mitigating factor ( Figures 2 and 3).

The model significantly predicts employee turnover (R ^2^ = 0.5552, F (3, 296) = 123.17, p < .001), with a significant interaction between toxic leadership and organizational commitment (B = 0.2124, p = .0088, 95% CI [0.0539, 0.3709]; R ^2^ change = 0.0105). This indicates that organizational commitment moderates the relationship, as toxic leadership’s impact on turnover increases with higher commitment. Although the moderation is statistically significant (p = 0.006; R ^2^ change = 0.050), its practical importance may be limited due to the modest variance explained. Surprisingly, committed employees may still leave under toxic leadership, emphasizing the need to address toxic behaviors irrespective of commitment levels ( Figures 6 and 7).

**Figure 4.**
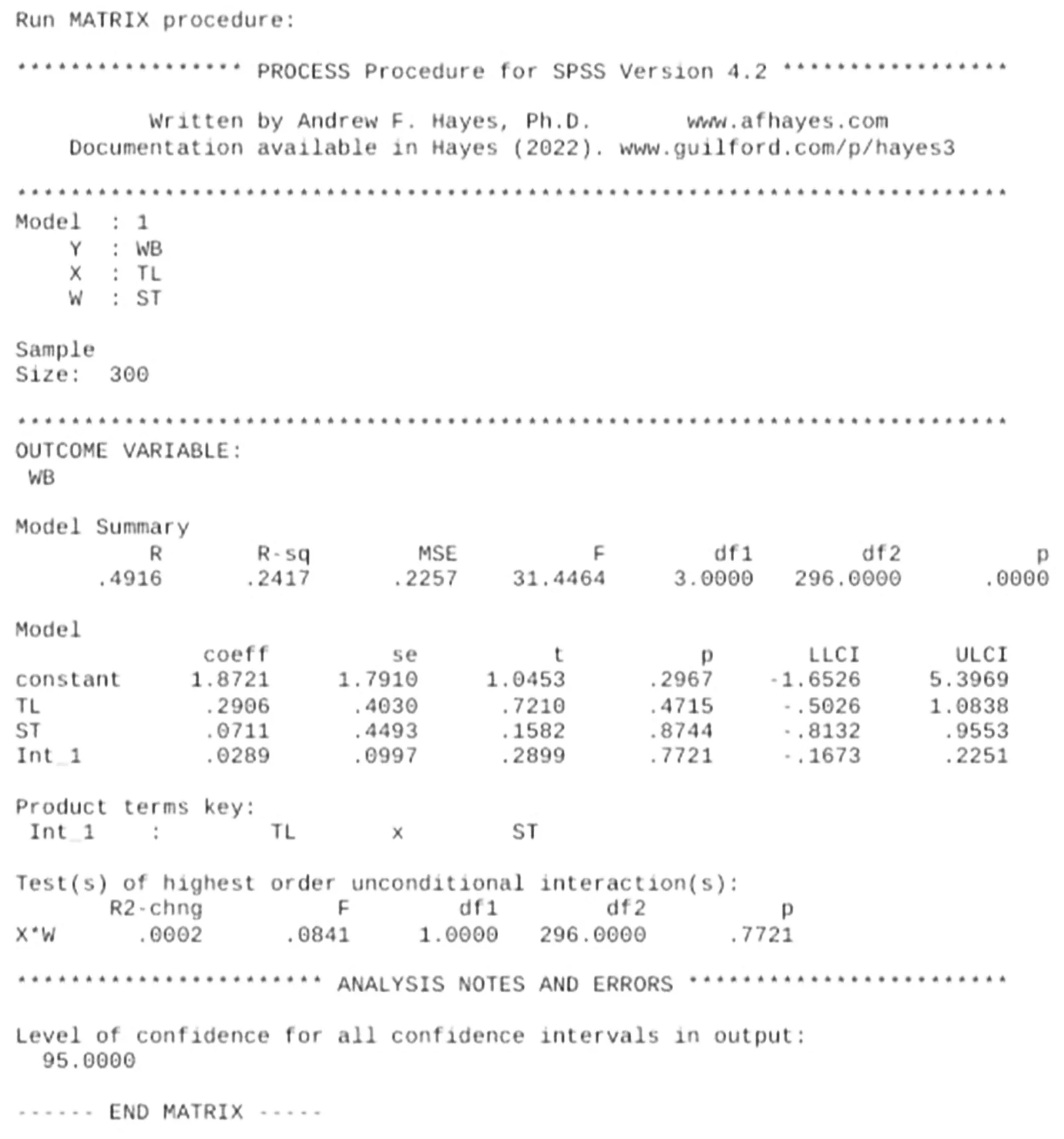
Regression output showing the moderating effect of shift timing on the relationship between toxic leadership and employee well-being.

**Figure 5.**
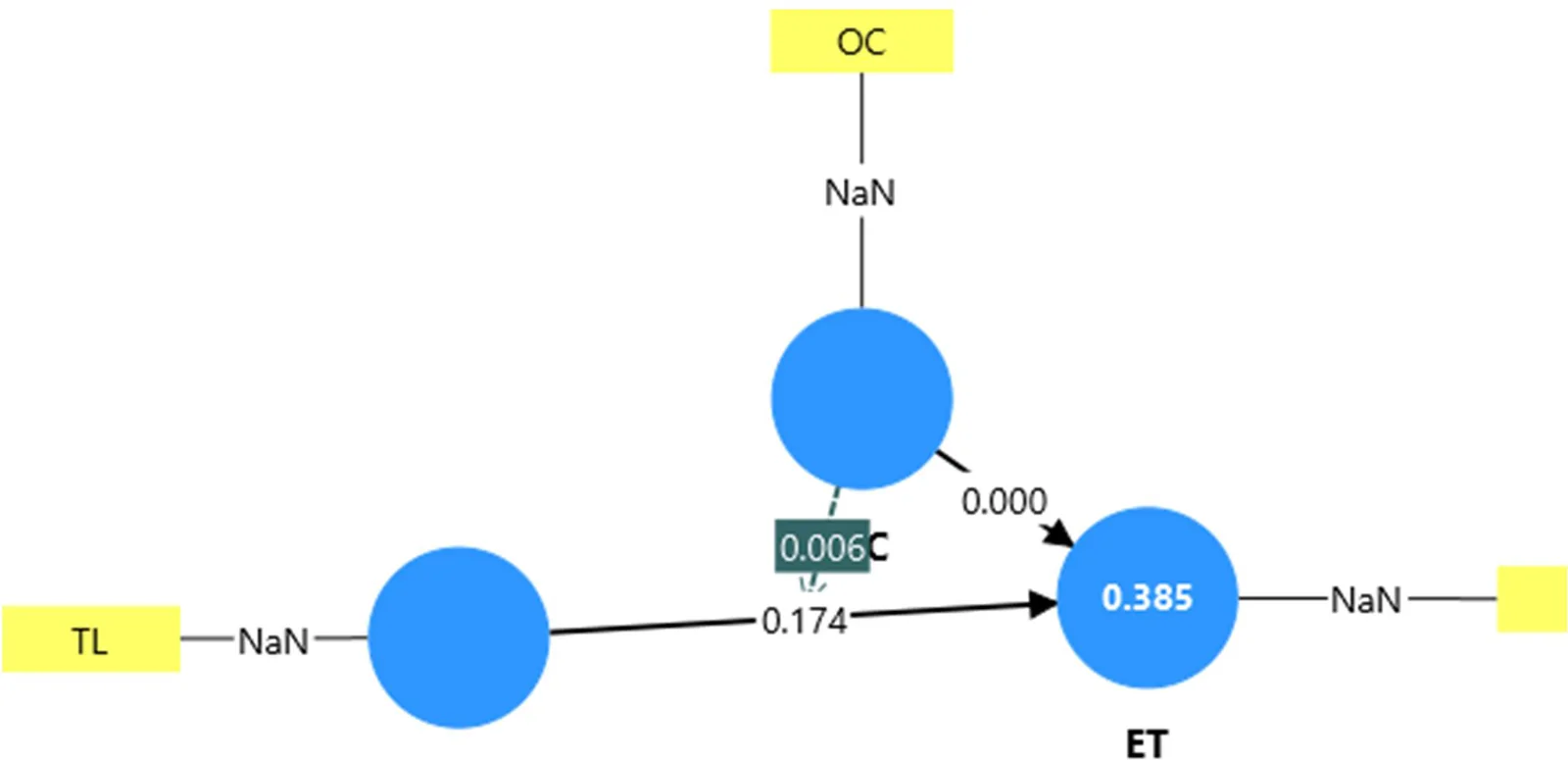
Interaction plot illustrating the conditional effect of shift timing on the relationship between toxic leadership and employee well-being.

**Figure 6.**
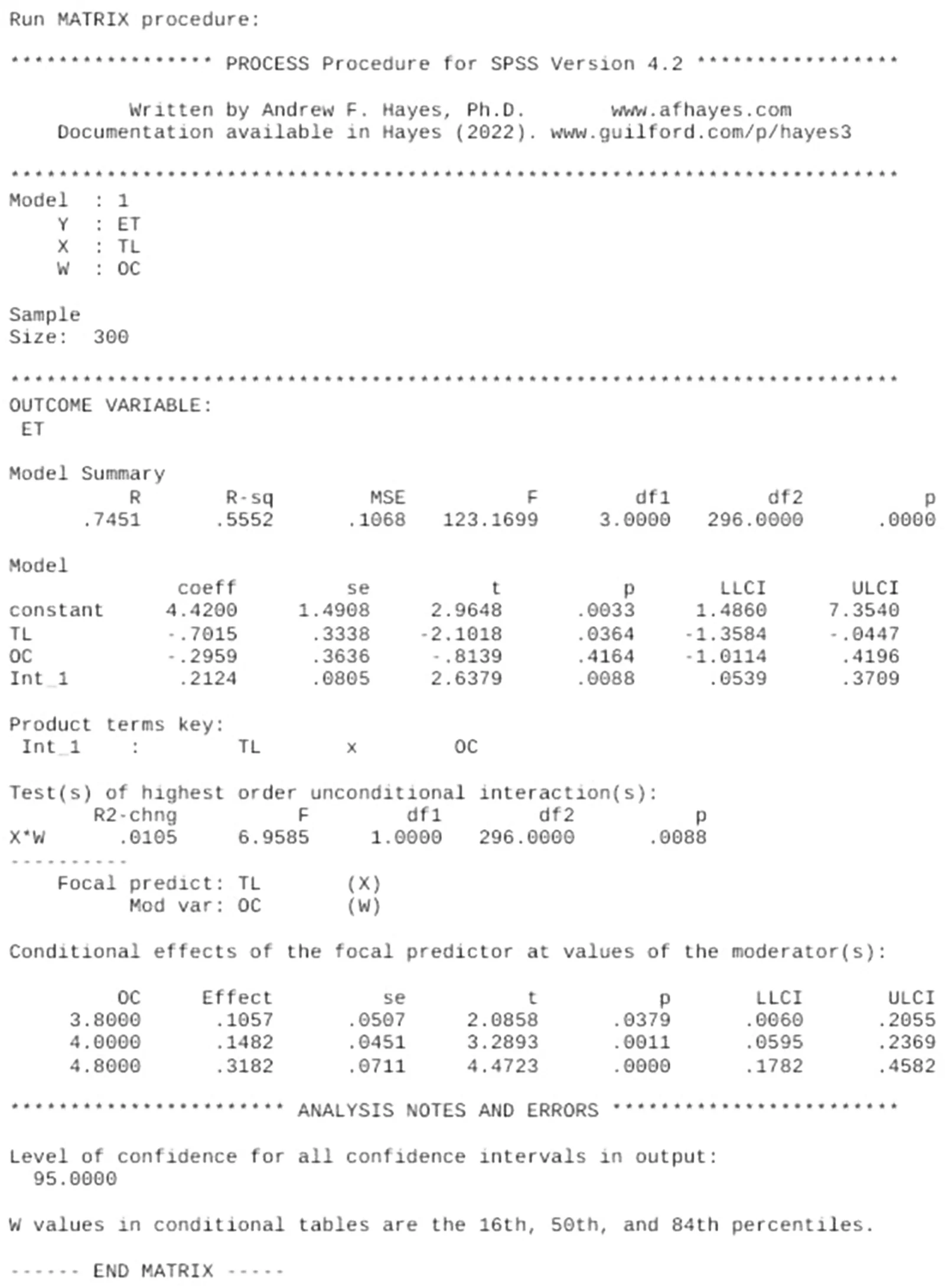
Regression output showing the relationship of toxic leadership on turnover.

**Figure 7.**
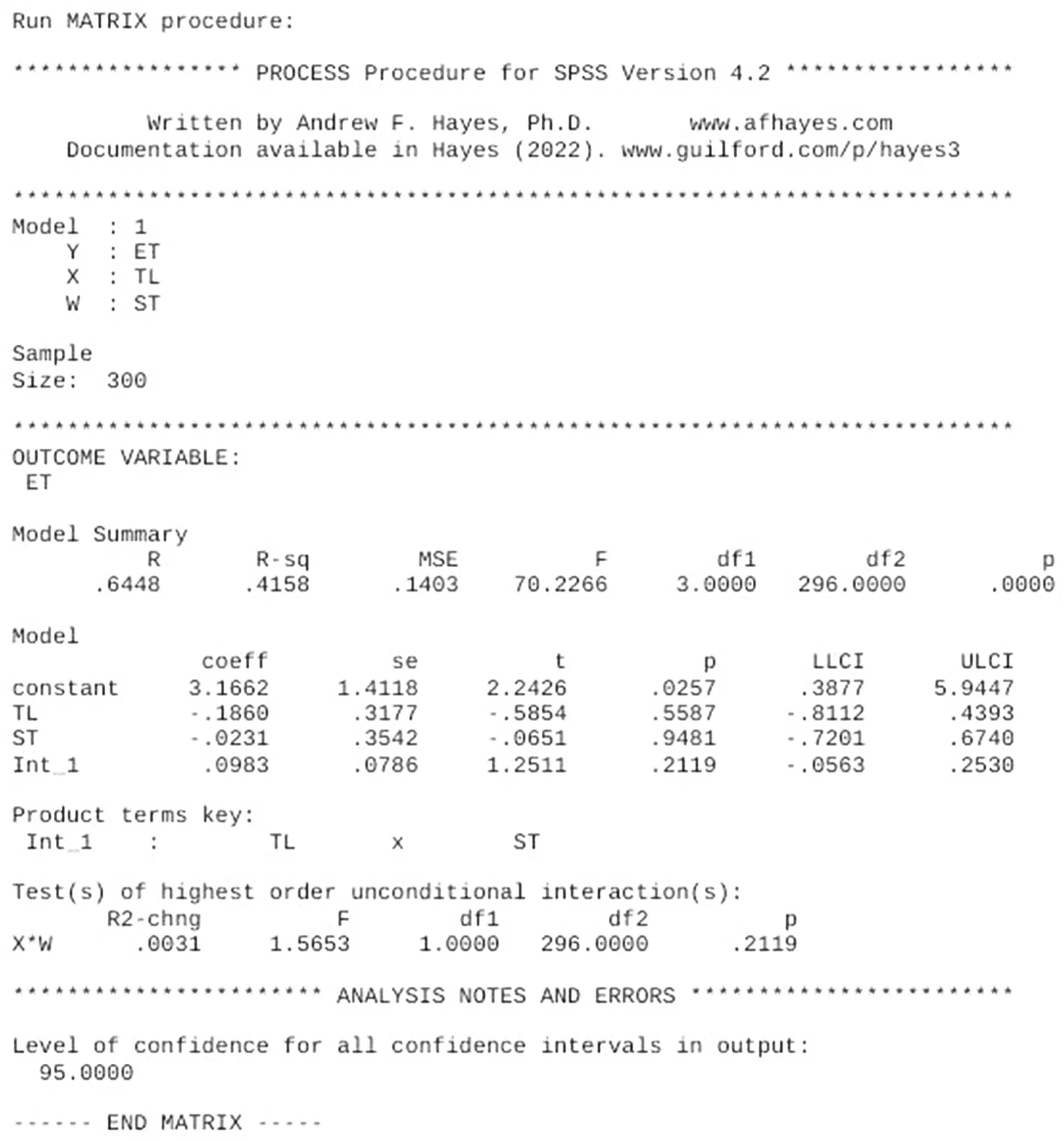
Regression output showing the relationship of organizational commitment on turnover.

The model significantly predicts employee turnover (R ^2^ = 0.4158, F (3, 296) = 70.23, p < .001), showing good overall fit. However, the interaction between toxic leadership and shift timings is weak, with an R ^2^ change of just 0.01 and a small coefficient (B = 0.165, p = .033), suggesting minimal practical significance. Although statistically significant, this moderation effect contributes little to explaining turnover ( Figures 4 and 5).

**Figure 8.**
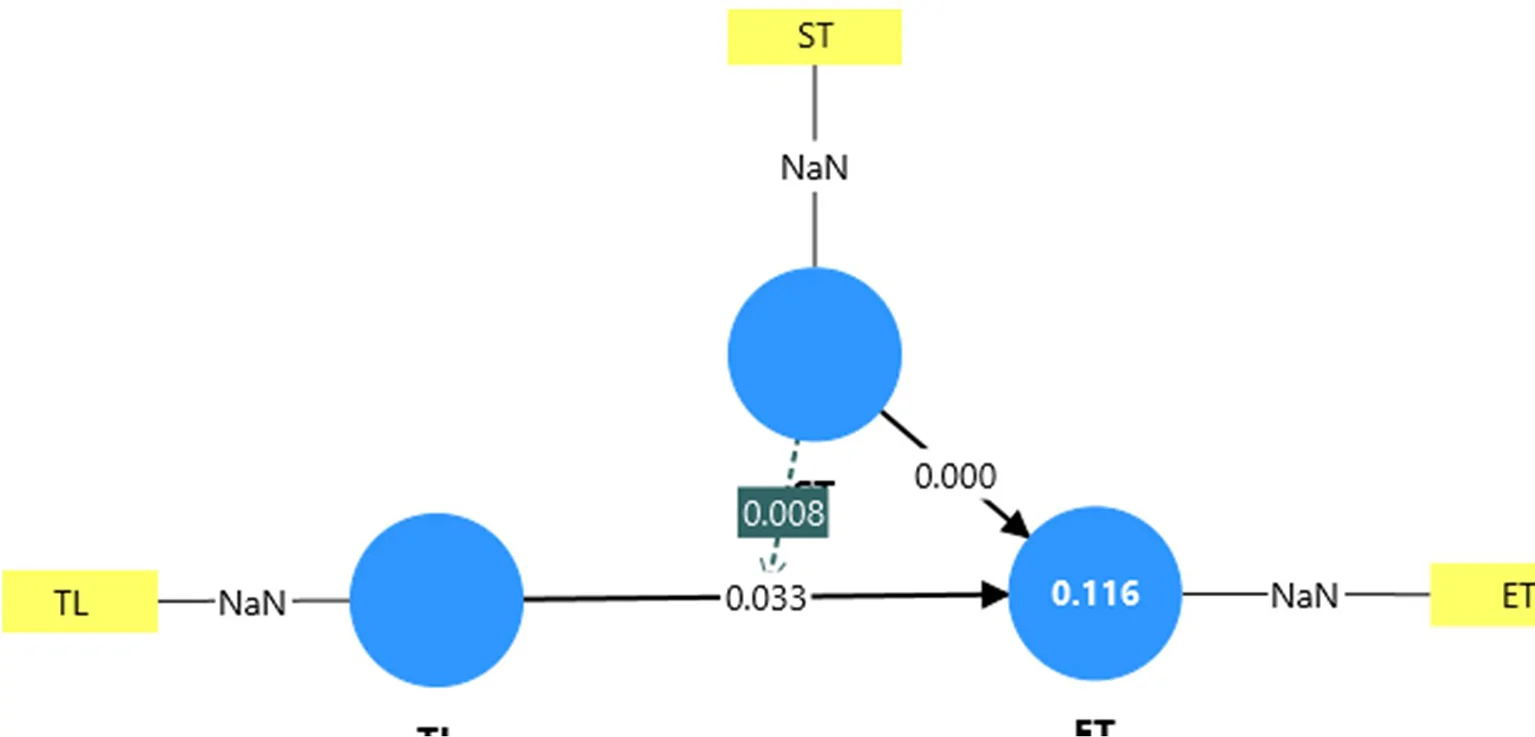
Interaction plot illustrating the limited moderating role of shift timings on turnover intention.

**Figure 9.**
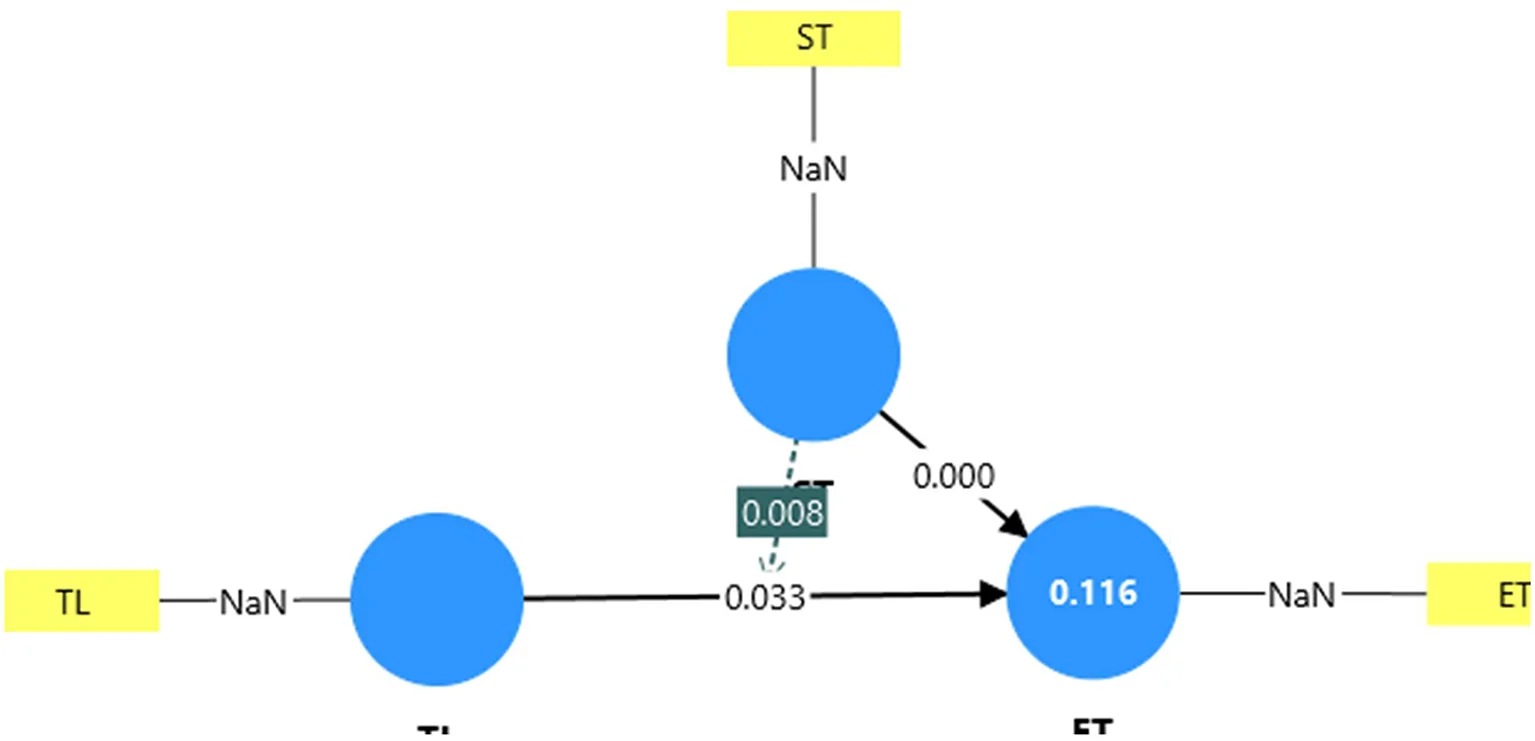
Interaction plot illustrating the limited moderating role of shift timings on turnover intention.

The main effects of toxic leadership (p = .5587) and shift timings (p = .9481) are not significant. Given the small effect size and potential inflation due to large sample size, the moderating role of shift timings should be interpreted cautiously ( Figures 8 and 9).

The model significantly predicts employee turnover (R ^2^ = 0.1841, F (3, 296) = 22.27, p < .001), but the interaction between toxic leadership and industry sector is not significant (B = 0.0382, p = .1479, 95% CI [–0.0136, 0.0901]), with a negligible R ^2^ change of 0.0058.

**Figure 10.**
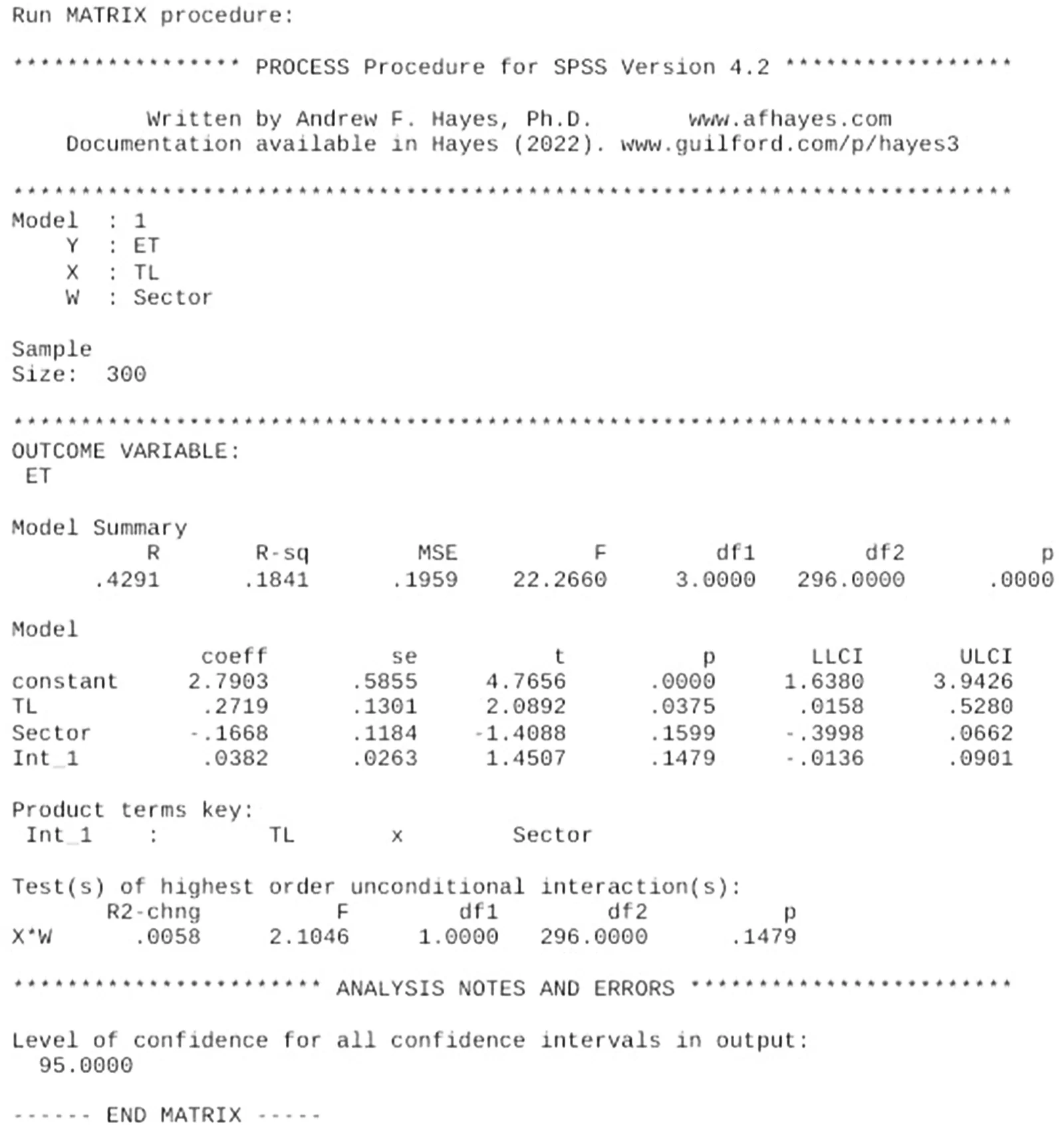
Regression output showing the relationship of toxic leadership’s impact differs across industry sectors.

The model significantly predicts employee turnover (R ^2^ = 0.1841, F (3, 296) = 22.27, p < .001), but the interaction between toxic leadership and industry sector is not significant (B = 0.0382, p = .1479, 95% CI [–0.0136, 0.0901]), with a negligible R ^2^ change of 0.0058. This indicates that sector does not moderate the relationship. The impact of toxic leadership on employee turnover (B = 0.2719, p = .0375) remains consistent across industries, suggesting toxic leadership is universally detrimental, regardless of sectoral context ( Figure 10).

**Figure 11.**
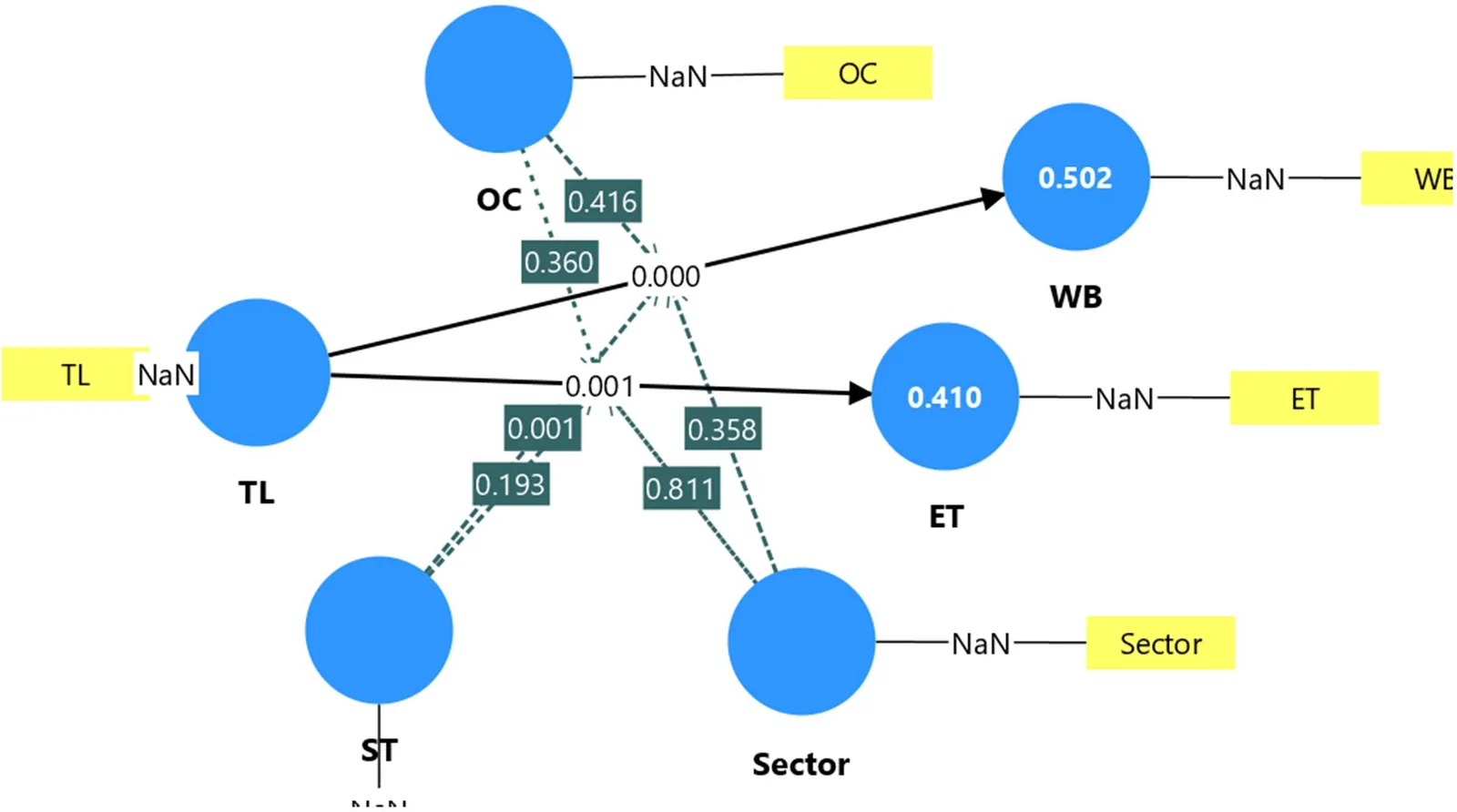
Structural equation model highlighting the results of both direct and moderating effects.

Figure 11 presents the structural equation model results highlighting both direct and moderating effects. The SEM analysis reveals that toxic leadership significantly impacts employee turnover (β = 0.151) and workplace behavior (β = 0.450). Organizational commitment (OC) strongly reduces turnover (β = 0.569) and slightly improves workplace behavior (β = 0.156). Shift timing (ST) and sector show weak or negligible effects. Interaction terms indicate OC moderate The SEM analysis reveals that toxic leadership significantly impacts employee turnover (β = 0.151) and workplace behavior (β = 0.450). Organizational commitment (OC) strongly reduces turnover (β = 0.569) and slightly improves workplace behavior (β = 0.156). Shift timing (ST) and sector show weak or negligible effects. Interaction terms indicate OC moderates the TL-ET link (β = 0.128), while ST and sector offer minimal moderation. R ^2^ values indicate good explanatory power for turnover (58.2%) and moderate for workplace behavior (37.3%). Reliability metrics (α > 0.73, AVE > 0.55) confirm construct validity. HTMT values < 0.85 confirm discriminant validity, and VIF < 3 rules out multicollinearity. Model fit is acceptable (SRMR = 0.081, NFI = 0.704). These findings highlight OC as a critical buffer against toxic leadership’s adverse outcomes.

### 5.1 Discussion of results

Hypothesis One reveals a moderate, possibly reversed, positive relationship between toxic leadership and employee well-being, indicating a potential measurement issue. Still, it supports prior research on the psychological harm of toxic leadership ( Labrague, 2024; Diab & Hassan, 2023). Psychological Contract, Conservation of Resources, and Social Exchange theories explain how unmet leadership expectations and resource depletion reduce well-being, emotional resilience, and job satisfaction.

Hypothesis Two confirms that toxic leadership predicts employee turnover, aligning with Gandolfi and Stone (2022) and Budak and Erdal (2022), who highlight reduced loyalty and morale. Theories of Social Exchange, Psychological Contract, and Conservation of Resources explain this as a response to trust breaches and resource depletion.

Unexpectedly, Hypotheses Three through Five show that neither organizational commitment nor shift timings mitigate the damage caused by toxic leadership. In fact, strong commitment may even worsen turnover outcomes when leadership is toxic. These results challenge previous assumptions ( Wolor et al., 2022; Ahmed et al., 2024) and further highlight how serious the resource and trust violations can become under toxic conditions ( Klahn and Male, 2024).

Hypothesis Six finds that shift timings neither influence the toxic leadership–turnover relationship nor hold independent significance, indicating consistent effects across schedules. This aligns with Ronnie (2024) and Octavian (2023), who emphasize the need for stable leadership across shifts. Psychological Contract Theory suggests fairness expectations remain constant, so violations impact all equally. Conservation of Resources Theory highlights that emotional exhaustion from toxic leadership is unaffected by timing, while Social Exchange Theory stresses that trust breaches harm employees regardless of shift. Hypothesis Seven shows no sector-based differences, supporting Lundqvist et al. (2025) and Semedo et al. (2022) in viewing toxic leadership as a universal issue.

The finding that organizational commitment worsens turnover under toxic leadership may be explained by the concept of moral injury, where highly committed employees experience deeper emotional conflict when their loyalty is betrayed. This breach intensifies feelings of disappointment and injustice, as suggested by Psychological Contract Theory. Loyal employees may expect respectful treatment, and when exposed to toxicity, their unmet expectations trigger stronger urges to leave. As Klahn and Male (2024) note, deep commitment can heighten sensitivity to toxic behavior, accelerating withdrawal.

## 6. Significance of the study

This study contributes to academic and practical understanding by revealing how toxic leadership affects employee well-being and turnover, with organizational commitment playing a complex role. The surprising positive link between toxic leadership and well-being calls for deeper exploration of measurement and leadership dynamics. Limited moderating effects of shift timing and industry sector highlight the widespread impact of toxic leadership. For leaders and HR professionals, the findings stress the need for targeted interventions and leadership development. Organizations that act on these insights can foster healthier work environments. Policymakers may also use the findings to guide ethical leadership standards and improve workplace practices across sectors.

## 7. Conclusion and recommendation

This study confirms that toxic leadership negatively affects employee well-being and turnover across all shifts and sectors. An unexpected positive link with well-being suggests a possible measurement issue needing further research. While organizational commitment reduces turnover and moderates its relationship with toxic leadership, it does not buffer well-being. Structural equation modeling identifies toxic leadership as a key predictor of negative outcomes. To mitigate its impact, organizations should prioritize leadership development focused on positive behaviors, conduct regular assessments, and gather employee feedback. Strengthening organizational commitment through supportive policies and ensuring consistent leadership practices can help improve retention and workplace climate across diverse settings.

## 8. Limitations and future research directions

The study’s reliance on a descriptive quantitative design limits the depth of understanding regarding participants lived experiences. Using a structured Likert scale survey may have constrained the range of responses and nuanced insights. Stratified random sampling enhanced representation but might not capture all sectoral or cultural variances within the UAE workforce.

Additionally, the positive association between toxic leadership and well-being, while statistically significant, raises concerns about potential interpretation bias or unaccounted mediating variables. The study was cross-sectional, limiting causal inference, and sector-specific contextual factors may have influenced outcomes. Organizational commitment moderated turnover but failed to buffer well-being, suggesting theory boundaries or unexplored mechanisms. SEM explained several relationships, but residual variances indicate additional influential factors not captured in the model.

Future research should consider a longitudinal or mixed-methods approach to better capture the dynamic and complex interplay between leadership behavior and employee outcomes over time. Qualitative interviews can offer deeper insights into employee perceptions of toxic leadership and organizational responses. Future studies should also explore cultural dimensions and additional moderators such as psychological safety, support systems, or team dynamics. Expanding the study to different countries or regions would enhance generalizability and allow comparison of cross-cultural leadership effects on employee well-being and turnover.

### Waived ethical approval statement

This study did not require prior ethical approval in accordance with the Institutional Ethics Committee (IEC) of Kasturba Medical College & Kasturba Hospital, Manipal (an associate hospital of MAHE, Manipal). As per *Appendix 2, Projects Exempt from Submission to IEC* (Registration No. ECR/146/Inst/KA/2013/RR-19; DHR Registration No. EC/NEW/INST/2022/KA/0042, dated 14 May 2024), research projects that are non-interventional, anonymous, and do not involve health, psychological, or social impact on human participants are exempt from review. Our study involved only voluntary, anonymous survey responses with no collection of personal or sensitive data and therefore qualified under this exemption.

All participants were provided with details of the research objectives and assured of the voluntary and confidential nature of their participation. Informed consent was obtained verbally prior to the completion of the survey.

### Informed consent

All participants provided verbal informed consent before participating in the survey. The purpose of the study, voluntary nature of participation, and data confidentiality were explained to all participants. Only participants who confirmed consent verbally were included in the study. Consent was obtained during the data collection period from November 2023 to January 2024.

## Data Availability

Zenodo. Understanding the Link Between Toxic Leadership and Employee Well-being: The Interplay of Employee Turnover, Organizational commitment, Shift timing & Industry Sector. https://doi.org/10.5281/zenodo.17776970 ( Abhishek S. 2025). This project contains the following underlying data:
• Color images.zip • Grey images.zip • Questionnaire. docx • Data.xlsx • SEM.xlsx •
Figure captions.docx Data are available under the terms of the Creative Commons Attribution 4.0 International license (CC-BY 4.0).
