# Understanding the Link Between Toxic Leadership and Employee Well-being: The Interplay of Employee Turnover, Organizational commitment, Shift timing & Industry Sector

**DOI:** 10.12688/f1000research.171577.2

**Published:** 2026-08-08

**Authors:** Abhishek Shukla, BR Santosh, Sangeetha Vinod

**Affiliations:** 1 Manipal Academy of Higher Education - Dubai Campus, Academic City, Dubai, United Arab Emirates; 2 Department of Commerce, Manipal Institute of Commerce, Finance and Economics, Manipal Academy of Higher Education, Manipal, India; 3 Manipal Academy of Higher Education - Dubai Campus, Academic City, Dubai, United Arab Emirates

**Keywords:** Toxic Leadership, Employee Well-being, Employee Turnover, Organizational Commitment, Shift Timing, Industry Sector, Quantitative Research, UAE Workforce

## Abstract

Background This study deals with the issue of toxic leadership and employees’ well-being, its effect on employees’ turnover among various industry sectors in the United Arab Emirates (UAE) and to gauge the moderating effect of organizational commitment, shift timing, and industry sector. Toxic leadership was consistently associated with adverse consequences for the employee but few studies have explored the relationship between both in a cross-sector, UAE-based context. Based on Social Exchange Theory, Psychological Contract Theory, and Conservation of Resources Theory, it elaborates an empirical model to make a holistic explanation of the impact of destructive leadership behaviors on employee attitude and the outcomes of the workplace.

Furthermore, the study was quantitative, and data were obtained from 300 workers from different industries in the UAE. Statistically, the data were analyzed by simple regression techniques and Structural Equation Modelling (SEM), which is used to find the direct and moderated relationships between variables. Furthermore, our findings indicated that toxic leadership was found to be a significant predictor of employee turnover and a significant determinant of employee well-being; however, the direction of the relationship was an unexpected result and deserves further consideration through the study of procedures and the operationalisations of constructs. Organizational commitment had a significant impact on the prevention of turnover and had limited buffering against toxic leadership. There were limited and negligible moderating influences for shift timing and industry sector.

Furthermore, the present study adds new contexts to the previous ones by adding workplace contexts and adds empirical evidence within the context of the UAE. The results also have practical consequences for employee support programmes, measures for employee retention and leadership development programmes in order to create a more sustainable working environment.

## 1. Introduction

In today’s competitive and ever-changing organizational environment, leadership plays a vital role in shaping employee experiences and outcomes. While positive leadership styles promote motivation, satisfaction, and performance, the negative effects of toxic leadership have gained increasing attention. Toxic leadership involves behaviors such as manipulation, hostility, and authoritarian control, which can harm both individual well-being and organizational effectiveness. As noted by Tanuwijaya and Jakaria (2022), toxic leadership fosters a hostile work climate that undermines psychological safety and morale. Iqbal et al. (2022) also highlight that such leadership increases stress and burnout, leading to a decline in overall employee well-being. These outcomes can be understood through Conservation of Resources Theory, which suggests that toxic environments drain employees’ emotional and psychological resources.

The impact of toxic leadership extends to increased employee turnover, as employees often choose to leave in search of healthier workplaces. According to Ofei et al. (2023), employees unable to manage the pressures of toxic leadership often resign. This aligns with Social Exchange Theory and Psychological Contract Theory, where broken expectations and imbalanced relationships drive employee exit. However, the effects of toxic leadership may vary. Nonehkaran et al. (2023) propose that organizational commitment can serve as a buffer, while Reyhanoglu and Akin (2022) identify shift timing as a possible moderator. This study investigates these dynamics to support better human resource strategies and leadership development.

## 2. Research aim and objectives

The study aims to understand the link between Toxic Leadership, Employee Well-being, Employee Turnover, Organizational commitment, Shift timing and Industry Sector.

The research addresses the following research objectives:

1. To assess the impact of Toxic Leadership on the Well-being of the employees.
2. To understand the impact of Toxic Leadership on Employee Turnover.
3. To examine the moderating roles of organizational commitment and shift timing between Toxic Leadership and Employee Well-being.
4. To examine the moderating roles of organizational commitment and shift timing between Toxic Leadership and Employee Turnover.

## 3. Literature review

### 3.1 Introduction

The work experience of employees and the performance of the organization have a strong relationship with the behavior of leadership. However, there was a growing interest in destructive leadership as scholars have been interested in positive leadership practices that have long-term effects. Toxic Leadership is a hostile, manipulative, self-interested, unpredictable and indifferent style of leadership behavior. Gandolfi and Stone (2022) defined toxic leadership as a repetitive type of negative behavior that negatively affects the functioning of the individual employees and the efficiency of the organization.

On the other hand, no psychological and behavioural impacts are experienced with ineffective leadership; toxic leaders with such negative leadership qualities bring them about. According to Octavian (2023), toxic leadership is important for creating an unhealthy organizational climate by instilling fear and restricting employee engagement in the decision-making process. Thus, such an environment mainly creates the condition and reduces employee resilience, which mainly encourages withdrawal and turnover. This also applies to the understanding of the influence of a toxic leader.

The researcher surveyed the abstracted academic journals, conference proceedings, technical reports, books, and other relevant publications to analyse and review the secondary literature sources. It emphasises the following subsections.

### 3.2 Toxic leadership and employee well-being

Employee Wellbeing involves the general psychological, emotional and work functioning in organizational settings. Waldiya (2023) explored that the nature of leadership is now well known to be a factor in employee well-being. Most of the previous research indicates that toxic leadership has a negative effect on employee well-being. Hadadian and Sayadpour (2018) discovered that there was a correlation between toxic leadership and stress, and that toxic leadership negatively affects job-related affective well-being. An individual who is continuously the subject of criticism, hostility or lack of support may show signs of emotional exhaustion and less satisfaction with work.

These observations have been further confirmed in recent years by further evidence. Toxic leadership was revealed to have adverse impacts on psychological outcomes as well as a reduction in employees’ perceptions of safety and support in the workplace ( Labrague, 2024). A poor job environment may also reduce staff trust in leadership and can have a negative impact on their emotional healing.

This relation can be explained with the help of the Conservation of Resources Theory. This theory posits that humans are working towards obtaining and maintaining key psychological resources throughout the process. Over time, emotional demands may be exacerbated, and capacities may be reduced, through the use of toxic leadership. This type of destructive leadership has a negative side because if an employee is subjected to this for a longer period of time, employee wellness can be affected, and can adjustment to the work.

However, if findings are not in line with a theory, care needs to be taken in interpreting the results. Other studies and research should be conducted to further examine the positive correlation between toxic leadership and employee well-being, as found in the current study. These arise for reasons of scale interpretation, operationalisation of the constructs or suppression in estimating the models and are basically a type of reverse coding. Thus, this must always be interpreted in terms of theories and evidence, and not to take it for granted that well-being is directly improving.

### 3.3 Toxic leadership and employee turnover

Soomro et al. (2024) examined that the employee turnover intention is conceptualized as a conscious choice of an employee to depart an organization. Employee turnover and succession are two predictors for quality leadership in an organization. Numerous studies have found that poisonous leadership leads to intend to quit. According to Bakkal et al. (2019), negative leadership contributes to events such as low satisfaction by employees and increased turnover rate tendency on their part. Disrespectful or aggressive interaction with the workers can cost for these workers to leave psychologically and remain constant.

Similarly, as stated by Hattab et al. (2022), the toxic leadership behavior is related to negative behavior including withdrawal and lessened organizational commitment. Psychological Contract Theory suggests that employees have certain expectations of what should transpire if they are to be treated fairly, supported, and respected. Thus, these expectations are not delivered as a result of unhelpful behaviours from the leaders then employees might react by dropping out and leaving the organisation. Turnover decisions can also be explained on the bases of Conservations of Resources Theory. Sometimes leaving organisations for them becomes too emotionally demanding, which may lead one to leave. The more the resources become depleted, the more vital, the more adaptive will be their leaving. Therefore, this perspective mainly explains why turnover often occurs among committed employees.

Leadership style remains central to shaping employee behavior across sectors. Constructive leadership enhances organizational performance, while toxic leadership diminishes engagement and productivity. Hattab et al. (2022), drawing on Psychological Contract Theory, showed that when employees perceive a breach in expected treatment due to toxic leadership, they are more likely to develop turnover intentions and engage in counterproductive behaviors.

Nunes and Palma-Moreira (2024) investigated how burnout mediates the relationship between toxic leadership and resignation intention. Toxic leadership was found to increase both burnout and turnover intention. Among Portuguese workers, burnout partially mediated this relationship through disengagement, while a complete mediation was observed among Angolan employees. These results further support the Conservation of Resources and Psychological Contract frameworks by showing how toxic leadership undermines employees’ mental energy and workplace expectations, leading to higher attrition.

### 3.4 Organizational commitment, shift timings, employee well-being, and employee turnover

Suárez-Albanchez et al. (2021) examined the influence of job-related safety and health regulations on employees’ commitment to both their roles and their organizations, and how this ultimately affects their intentions to resign. The study established a negative relationship between workplace safety and health policies and the intention to leave, along with a positive relationship between those regulations and both job involvement and organizational commitment. In addition, the results revealed that employees’ job performance and organizational loyalty are negatively linked to their desire to exit, suggesting that improved regulatory frameworks enhance engagement and reduce resignation tendencies.

In the hospitality sector, Dorta-Afonso et al. (2021) explored the pathways through which high performance work systems affect employees’ satisfaction and psychological well-being. Their investigation confirmed that such systems directly improve job satisfaction, motivation, commitment to the organization, and overall quality of life. Furthermore, organizational commitment and motivation significantly influence job satisfaction, which in turn contributes positively to life quality. The study also found that individual job performance is greatly enhanced when employees report high levels of satisfaction and well-being, highlighting the essential role of supportive workplace systems in performance outcomes.

Focusing on the unique pressures during the COVID-19 crisis, Serhan et al. (2022) investigated how organizational commitment influences the turnover intentions of Islamic bank employees. The results revealed significant associations among emotional, normative, and continuance commitment, organizational loyalty, individual differences, and resignation intentions. Each dimension of organizational commitment was shown to reduce the desire to leave the job. Furthermore, the relationship between commitment and turnover intention was moderated by both employee-specific traits and perceived organizational performance, suggesting that retention strategies must be tailored to individual and contextual factors.

Suter et al. (2020) evaluated how employees in high-intensity mental health environments respond to a newly introduced 12-hour work shift from a well-being perspective. The findings indicated that such extended shift structures produce varied effects on worker well-being, with notably harmful consequences in high-stress and complex organizational contexts. The research emphasized the importance of worker autonomy and flexibility for maintaining well-being and staff retention, particularly in aging and competitive labor markets. Consequently, the study recommended that imposed 12-hour shift systems should be reconsidered or discontinued to preserve employee health.

Applying the Guidelines for Worker Well-Being, McHugh et al. (2020) investigated how manufacturing employees perceive the effects of shift work. Participants reported that shift work had a detrimental influence on well-being in four out of five framework categories. However, in the area of workplace culture and employment norms, some workers viewed shift schedules as necessary, fair, and financially beneficial. These mixed responses underscore that the impact of shift work is highly dependent on contextual and perceptual factors, reinforcing the need for nuanced organizational policies that account for both risks and benefits of such employment structures.

### 3.5 Integrated theoretical framework

The present study has adopted a multi-part theoretical approach by incorporating Social Exchange Theory (SET), Psychological Contract Theory (PCT) and Conservation of Resources (COR) Theory in order to explain different mechanisms through which toxic leadership can have an impact on employee well-being and turnover intention. Cropanzano et al. (2017) have said that workplace relationships are conducted in a manner of exchangeable service where employees look for fair treatment, respect and support from the organisation. These reciprocal exchanges decline when the leaders act in manipulative & hostile ways, which in turn diminishes employee trust and their desire to return favours related to positive OBs. For this reason, the theoretical bases of H1 and H2 are based on Social Exchange Theory, which investigates the relationship between the toxic leadership and employees’ well-being and intention to leave.

Psychological Contract Theory complements Social Exchange Theory by explaining employees’ reactions when organisations fail to fulfil their perceived obligations and expectations. According to Hattab et al. (2022), destructive leadership violates employees’ psychological contract, causing psychological contract violations, employees’ dissatisfaction, lower organisational commitment, and high turnover Intention. In line with this theory, the main support will be for H2, H3 and H4 which is the belief that organisational commitment will affect employees’ reactions to toxic leadership.

Conservation of Resources Theory further explains that employees strive to preserve valuable emotional and psychological resources, which become depleted when they are exposed to toxic leadership behaviours. These resources continue to diminish over time as a result of toxic leadership and lead to greater emotional exhaustion and lower employee well-being ( Hobfoll et al., 2018).

Furthermore, the extent of resource depletion may vary according to employees’ work schedules and organisational environments, providing the theoretical rationale for Hypotheses H5, H6, and H7. These three theories provide complementary rather than competing explanations for the mechanisms through which toxic leadership influences employee outcomes. However, if SET is used, it can explain deteriorating exchange relationships, if PCT is used, it can explain misaligned employee’s expectations, and if COR Theory is used, it can explain depletion of psychological resources thus integrating all of them for the purposes of this study.

### 3.6 Research gap

Despite an increasing body of research on the subject of toxic leadership, there are several important areas that are underserved. Studies of employee well-being and studies of employees’ intention to leave their current organizations have focused on these two aspects separately, and relatively few studies have studied both at the same time within a single, coherent study. Consequently, the combined psychological and behavioural consequences of toxic leadership remain insufficiently understood.

Second, the focus of current research is mainly direct effects of toxic leadership with minimal consideration to contextual factors that can have an impact on these relationships. However, relatively few empirical studies have examined the potential moderating effects of work organization factors such as organizational commitment, shift timing and industry sector, though the potentially important ways in which organizational context might influence the reactions of workers to destructive leadership behaviors are suggested.

Furthermore, most empirical evidence has been generated in Western countries or within single-industry settings, limiting the generalizability of findings to multicultural labour markets such as the UAE.

In light of these gaps, this study fills these gaps by concurrently analyzing both employee well-being and turn intentions, examines the role of organizational commitment, shift timing and the role of industry sector as moderators and delivers empirical evidence from employees working in a variety of industry sectors in the UAE. The study brings together these variables in one theoretical framework, thus filling the gap in the existing knowledge about toxic leadership in multicultural work environments.

### 3.7 Hypothesis development

Drawing upon Social Exchange Theory, Psychological Contract Theory, and Conservation of Resources Theory, this study develops seven hypotheses to examine the relationships among toxic leadership, employee well-being, turnover intention, organisational commitment, shift timing, and industry sector.

The Social Exchange Theory describes how negative and unfair relationships within the workplace can lead to negative relationships with employees as well. Toxic leadership may make people feel less supported by the organisation and be psychologically more affected negatively. Conservation of Resources (COR) Theory further implies that employees’ emotional and psychological resources are engrossed, even if not completely exhausted, by the challenge of adversarial leadership and consequently, employees’ well-being is negatively impacted by this suboptimal condition. Therefore, this work has been conducted with the assumption of toxic leadership impacting the well-being of an employee, and the studies generally have affirmed that belief.

H01: There is no significant relation between toxic leadership and the well-being of staff.

H1: Employee health and wellness can be affected by the leadership of leaders.

There are two types of explanation of employee turnover, which are reciprocal exchange and psychological exchange. Employees who experience hostility, manipulation, or inadequate organisational support are more likely to consider leaving their organisation. As discussed by Gandolfi and Stone (2022), toxic leadership can have detrimental effects on employee commitment and employee withdrawal tendency.

H02: There is no statistically significant relationship between toxic leadership and turnover intention.

H2: Toxic leadership mainly affects the employee turnover intention.

Organisational commitment may influence how employees respond to toxic leadership by strengthening their attachment to the organisation despite adverse workplace experiences. Previous studies have reported inconsistent findings regarding whether highly committed employees are better able to withstand negative leadership experiences.

H03: Organisational commitment does not significantly moderate the relationship between toxic leadership and employee well-being.

H3: Toxic leadership and the well-being of employees, there is a mediating role of the organizational commitment.

Organizational commitment can also influence relationship outcomes of employee turnover if leadership is destructive, as this can help to cultivate deep attachment as well as great disappointment when expectations are not met.

H04: There is no moderation between the relationship of toxic leadership and employee turnover intention by organizational commitment.

H4: The organizational commitment is a moderator between toxic leadership and employee intention to quit the organization.

Work arrangements might impact employees’ interaction with leaders and other pressures at work. Leadership relationships could vary according to the timing of a worker’s work and this could affect their well-being.

H05: There is no interaction between the timing hypothesis and the toxic leadership and employee well-being.

H5: Shift timing mainly moderates the linkage between employee well-being and toxic leadership.

Employee responses to shifts in employee turnover might also relate to shift timing, as it can impact employee experiences of stress and organizational support.

H06: Shift timing does not significantly moderate the relationship between toxic leadership and turnover intention.

H6: Shift timing moderates the relationship between toxic leadership and employees’ turnover intention.

Expectations on organisations and working procedures differ between business sectors, and employees have varying expectations. The differences are very different and can impact how toxic leadership behaviours are felt by employees.

H07: Industry sector does not show the connection between employee turnover intention and toxic leadership.

H7: Industry sector moderates the connection between employee turnover intention and toxic leadership.

### 3.8 Conceptual framework

**Figure 1.**
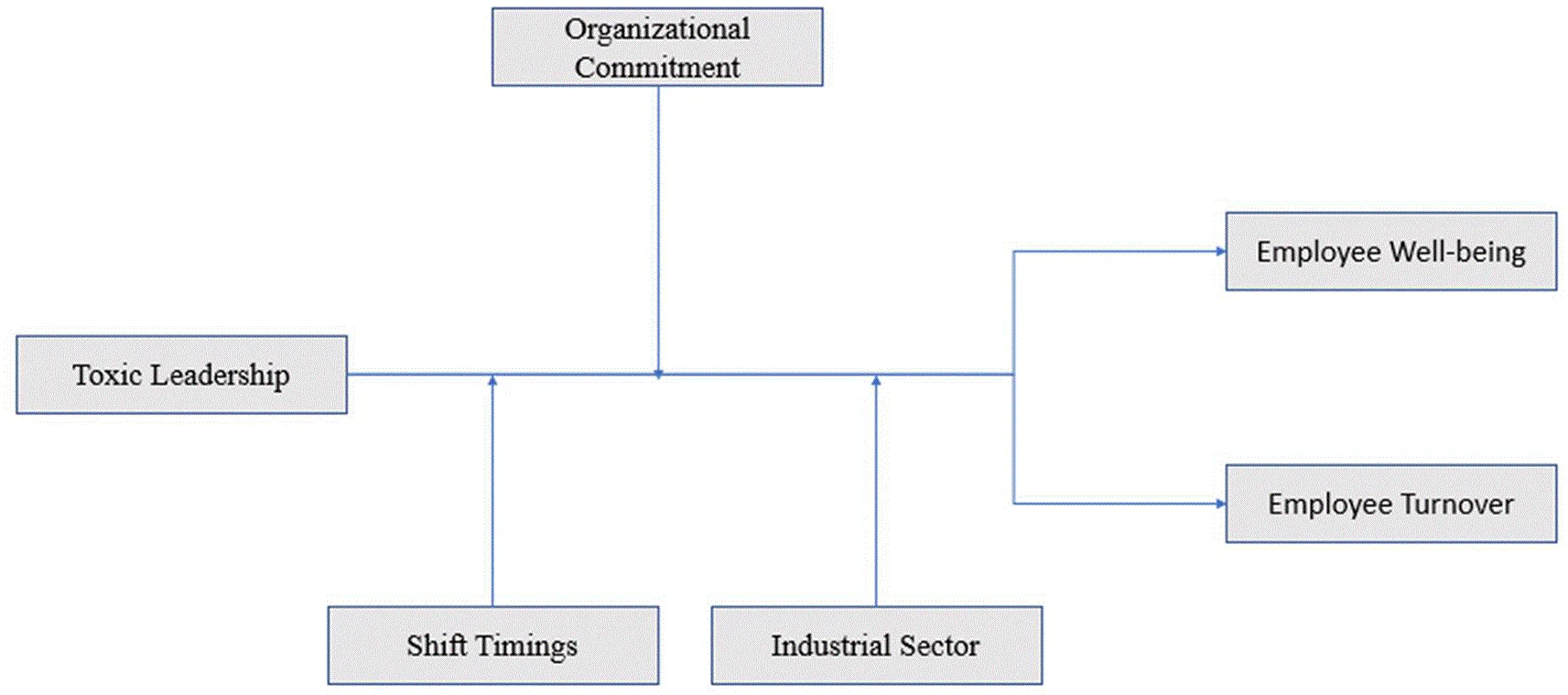
Conceptual framework showing the relationship between the dependent, independent, moderator and mediator variables.

The relationships among variables are illustrated in the conceptual model in Figure 1.

## 4. Research methodology

This study used a cross-sectional quantitative research design, focusing on the relationship between toxic leadership, employee well-being and employee turnover in various sectors in the United Arab Emirates (UAE). A quantitative approach enabled objective examination of the relationships among the study variables and responses of employees can be interpreted. Ghanad (2023) suggested that the quantitative methods can be used to find patterns and test the possible relationship.

Structured self-administered questionnaires were filled out by data collected from employees from various sectors. Stratified random sampling was used to increase representation by industry and to minimise sampling imbalance. According to Rahman et al. (2022), the strength of probability-based sampling is that it enhances the generalizability and thus minimises the selection bias.

Inclusion or exclusion from the sample necessitated that respondents be in a job role and working within a formal organizational leadership system. The data collection process was voluntary and anonymous. Ethical safeguards included informed consent, confidentiality, anonymity, and the removal of identifying information to minimise participant risk and encourage honest responses.

This study adopts a positivist research paradigm, which emphasizes objectivity, measurement, and hypothesis testing, allowing for empirical assessment of relationships among variables ( Junjie & Yingxin, 2022; Sanchez et al., 2023). A quantitative research approach was chosen to ensure precision and generalizability through numerical data analysis ( Jamieson et al., 2023; Ghanad, 2023). The research follows a descriptive research design, which is suitable for systematically describing the nature and strength of relationships among toxic leadership, employee well-being, turnover, organizational commitment, shift timing, and industry sector ( Taherdoost, 2022; Furidha, 2023). Data was collected using a structured Likert-scale survey, which is widely used for measuring attitudes and perceptions in organizational settings ( Jebb et al., 2021; South et al., 2022). The target population includes all employees working across various sectors in the UAE, ensuring a broad and inclusive understanding of workplace dynamics. The overall methodology of the research process is outlined in Table 1.

**Table 1.** RM flow chart.

	Initial
My supervisor/leader demeans or belittles the team frequently.	1.000
Supervisors in our organisation take credit for work done by subordinates.	1.000
Our supervisor shows favouritism toward specific employees, creating a toxic work environment.	1.000
Our supervisor/leader blames others for their own mistakes or failures.	1.000
Our supervisor is more concerned with their personal gain compared to team success or employee well-being.	1.000
I feel emotionally and mentally healthy at my workplace.	1.000
I feel enthusiastic and motivated to perform my day-to-day tasks.	1.000
I have a good work-life balance that allows me to rest and give time to my family.	1.000
I feel that my work contributes to my personal growth and fulfillment.	1.000
I can openly communicate my concerns or challenges at work.	1.000
I frequently think about leaving my current job.	1.000
If I received a better offer, I would leave this company immediately.	1.000
The lack of career growth opportunities often motivates me to consider leaving.	1.000
Poor leadership in my department has made me consider quitting.	1.000
I feel my contributions are not recognised, which affects my desire to stay in the organisation.	1.000
I feel a strong sense of belonging to my company.	1.000
I feel emotionally attached to this company.	1.000
It would be very hard for me to leave this organisation right now, even if I wanted to.	1.000
In my opinion, loyalty to one’s organisation is important and should be maintained.	1.000
I am committed to the success and goals of this company.	1.000
My shift timing lets me maintain a healthy work-life balance.	1.000
My existing shift does not interfere with my social or family life.	1.000
My job satisfaction is affected by the timing of my shift.	1.000
I feel well-rested and alert throughout my shift.	1.000
I am satisfied with my existing shift timing.	1.000

Extraction Method: Principal Component Analysis.

Stratified random sampling was used, with 300 valid questionnaires being finally analysed. Stratified sampling has been proposed by Rahman et al. (2022) as a better way to improve sample representativeness, as there is proportional representation of different organisational groups. So, respondents are representative of the UAE’s health sector, manufacturing, retail, education, hospitality sector, logistics sector, financial services and other services sectors.

About 350 questionnaires were distributed, and 300 were found to be usable, with an 85.7% response rate for usable questionnaires. Baruch and Holtom (2008) noted that a greater than 70% response rate is a good rule of thumb for organisational survey research.

The participants had to have organisational experience for a period of at least six months, to be full-time employees and have a direct reporting relationship with their immediate supervisors. The individuals who were not considered temporary staff, interns, freelancers and senior executives without reporting managers were excluded, as the perception of toxic leadership was assessed to make the results consistent.

**Ethical considerations**

The research did not require formal ethical approval as this was an anonymous, non-interventional questionnaire survey, but extensive ethical safeguards were put in place during the research process. No one was forced to participate, and respondents had the freedom to stop at any time without any repercussions. Voluntary participation is a key rule of good human research, according to the World Medical Association (2013).

Personally identifying information was not collected, and responses were anonymised before statistical analysis. Electronic data were securely stored by having password-protected systems that could only be accessed by the research team. Partaking participants were also notified that they might not have to reply to any question that might make them uneasy, as this survey seeks to examine sensitive experiences at work ( British Psychological Society, 2021).

**Informed consent**

All participants provided verbal informed consent prior to completing the survey. Participants were comfortable for the survey but were particular on verbal consent due to cultural sensitivity, anonymity concerns, and the non-intrusive nature of the study. Hence, this was opted for the study. Participation was voluntary, and responses were anonymous. No personal identifiers were collected.

### 4.1 Statistical analysis

Data analysis was performed using regression analysis and Structural Equation Modeling (SEM) to look at the direct and moderating relationship between variables, namely, toxic leadership, employee well-being, employee turnover, organizational commitment, shift timing and industry sector. To test individual hypotheses and moderation effects, regression analysis was used, while the integrated structural relationships of constructs were tested using SEM. Attwal and Singh (2024) stated that such an analysis can be carried out with the goal of testing a hypothesis or evaluating the overall model.

Since multiple direct and moderating relationships were examined together, the study adopted Structural Equation Modelling (SEM) technique in conjunction with Partial Least Squares estimation procedure in analysing the data using SPSS Amos computer software. The study used SPSS Amos computer software program with Structural Equation Modelling (SEM) technique and Partial Least Squares estimation procedure because the study examined multiple direct and moderating relationships. When prediction and theory development are the top goals in research under complex structural relationships, PLS-SEM was recommended by Hair et al. (2022). Multiple regression analysis was followed after operational testing of individual hypotheses and validated using a semi-structural model in the direction of a structural model SEM. For the evaluation of model adequacy, SRMR, NFI, CFI, TLI and RMSEA were used. Kline (2023) proposed that rather than using a single fit statistic, a number of fit indices could be used jointly. The model fit measured by the SRMR was satisfactory, but the relatively low NFI suggests giving the results from this structural model appropriate interpretation.

Table 2 presents the communalities analysis showing total variance explained across variables. The communalities analysis using Principal Component Analysis reveals that all items initially have communalities of 1.000, indicating that each variable’s total variance is considered in the extraction process. However, since only initial communalities are provided and no extracted values are shown, it is not possible to assess how much variance in each item is explained by the underlying components after extraction. Initial communalities of 1.000 are typical before factor extraction, as they represent the assumption that each item’s variance is fully accounted for. To interpret the effectiveness of the factor solution, the extracted communalities would be required to evaluate shared variance among the variables. Measurement reliability and construct validity were completed before hypothesis testing. Cronbach’s α coefficient and composite reliability (CR) were used to assess for internal consistency. Cronbach’s alphas for the constructs were greater than 0.73, which is the recommended threshold, and thus the instruments were judged to have a high degree of reliability. The values of composite reliability stayed within recommended limits thus ensuring the internal consistency of the measure instrument.

**Table 2.** Communalities analysis.

Total Variance Explained						
Component	Initial Eigenvalues			Rotation Sums of Squared Loadings		
	Total	% of Variance	Cumulative %	Total	% of Variance	Cumulative %
1	8.531	34.126	34.126	5.684	22.735	22.735
2	4.722	18.889	53.015	4.863	19.45	42.186
3	3.724	14.895	67.91	3.506	14.025	56.21
4	2.164	8.658	76.568	3.211	12.845	69.055
5	1.317	5.266	81.834	2.463	9.851	78.907
6	1.046	4.184	86.018	1.778	7.112	86.018
7	0.912	3.647	89.665			
8	0.755	3.021	92.687			
9	0.577	2.308	94.995			
10	0.347	1.387	96.381			
11	0.237	0.947	97.329			
12	0.199	0.794	98.123			
13	0.164	0.656	98.779			
14	0.11	0.441	99.22			
15	0.074	0.295	99.515			
16	0.066	0.265	99.78			
17	0.049	0.196	99.976			
18	0.006	0.024	100			
19	1.26E-16	5.03E-16	100			
20	6.80E-17	2.72E-16	100			
21	8.88E-18	3.55E-17	100			
22	-3.88E-33	-1.55E-32	100			
23	-2.30E-17	-9.18E-17	100			
24	-1.06E-16	-4.24E-16	100			
25	-2.20E-16	-8.81E-16	100			

**Measurement model Assessment**

The reliability of the measures were evaluated by Cronbach’s α, Composite Reliability (CR), Average Variance Extracted (AVE), and Heterotrait-Monotrait Ratio (HTMT). Hair et al. (2022) suggest that values of Cronbach’s alpha > 0.70 indicate acceptable internal consistency, while Composite Reliability values > 0.70 are considered to reflect an adequate level of internal consistency. For all constructs, reliability was above these recommended levels, which is considered good measurement reliability. Construct dimensionality was examined using AVE, with all the values of constructs being greater than the recommended value of 0.50, which provided convergent validity. Henseler et al. (2015) suggested that when the value of HTMT is below 0.85, it means that the latent construct has a satisfactory degree of discriminant validity. In light of this, the measurement model has acceptable construct validity and reliability.

**Table:** 

Construct	Cronbach α	Composite reliability	AVE
Toxic Leadership	0.912	0.926	0.714
Employee Well-being	0.893	0.917	0.689
Turnover Intention	0.901	0.921	0.701
Organisational Commitment	0.885	0.908	0.663
Shift Timing	0.876	0.901	0.645

As shown in Table 3, six components accounted for over 86% of the total variance. The communalities analysis indicates that all 25 items initially had communalities of 1.000, reflecting that each item’s total variance was included before factor extraction. Using Principal Component Analysis, six components were extracted with eigenvalues greater than 1. Together, these components explain 86.02 percent of the total variance. The rotation sums of squared loadings reveal that the first component explains 22.74 percent, the second 19.45 percent, and the third 14.03 percent of the variance, cumulatively reaching 56.21 percent. This suggests a strong factor structure with meaningful underlying dimensions. However, the actual communalities after extraction would be needed to assess how well each item is represented. The Average Variance Extracted (AVE) was used to assess the convergent validity. All the AVE values were above the 0.55 threshold thus suggesting that the latent constructs accounted for more than 55 % of the variance in their observed indicators. A score within the acceptable range eases the worries about the measurement inconsistency, adds Watkins (2021), while improving construct interpretation.

**Table 3.** TVA analysis.

Rotated Component Matrix						
	Component					
	1	2	3	4	5	6
My supervisor/leader demeans or belittles the team frequently.		0.969				
Supervisors in our organisation take credit for work done by subordinates.		0.877				
Our supervisor shows favouritism toward specific employees, creating a toxic work environment.	0.95					
Our supervisor/leader blames others for their own mistakes or failures.			0.958			
Our supervisor is more concerned with their personal gain compared to team success or employee well-being.			0.958			
I feel emotionally and mentally healthy at my workplace.	0.514					
I feel enthusiastic and motivated to perform my day-to-day tasks.	0.95					
I have a good work-life balance that allows me to rest and give time to my family.	0.765					
I feel that my work contributes to my personal growth and fulfillment.	0.829					
I can openly communicate my concerns or challenges at work.					0.879	
I frequently think about leaving my current job.					0.793	
If I received a better offer, I would leave this company immediately.				0.83		
The lack of career growth opportunities often motivates me to consider leaving.				0.691		
Poor leadership in my department has made me consider quitting.				0.815		
I feel my contributions are not recognised, which affects my desire to stay in the organisation.				0.839		
I feel a strong sense of belonging to my company.					0.667	0.531
I feel emotionally attached to this company.						0.77
It would be very hard for me to leave this organisation right now, even if I wanted to.						0.693
In my opinion, loyalty to one’s organisation is important and should be maintained.	0.66					
I am committed to the success and goals of this company.	0.56					
My shift timing lets me maintain a healthy work-life balance.		0.969				
My existing shift does not interfere with my social or family life.		0.877				
My job satisfaction is affected by the timing of my shift.	0.95					
I feel well-rested and alert throughout my shift.			0.958			
I am satisfied with my existing shift timing.		0.969				

Extraction Method: Principal Component Analysis.

Rotation Method: Varimax with Kaiser Normalization.

Table 4 displays the rotated component matrix with distinct item loadings across six components. The rotated component matrix from the Principal Component Analysis with Varimax rotation reveals a clear factor structure, with items strongly loading onto six distinct components. Items related to toxic leadership, employee well-being, turnover intentions, organizational commitment, and shift satisfaction load highly on separate components, indicating well-defined constructs. For instance, toxic leadership items load above 0.95 on Component 1, while turnover-related items load strongly on Component 3. Shift-related items also show high loadings on Component 6. This structure supports the validity of the questionnaire in capturing multiple dimensions. The strong loadings across items indicate that the extracted components effectively explain the shared variance among variables. Heterotrait–monotrait ratio (HTMT) was used to check the discriminant validity. The content of the items was also satisfactory with respect to conceptual differentiation, and the factor loadings for the outer items in the HTMT were below 0.85. Additionally, the multicollinearity of the variables was checked with the help of Variance Inflation Factor (VIF) which showed values of less than 3, suggesting that there was no significant overlapping in variables used for the estimations.

**Table 4.** Rotated component matrix analysis.

Model Summary				
Model	R	R Square	Adjusted R Square	Std. Error of the Estimate
1	.445 ^a^	0.198	0.195	0.48701

Anova						
Model		Sum of Squares	df	Mean Square	F	Sig.
1	Regression	17.424	1	17.424	73.461	.000 ^b^
	Residual	70.68	298	0.237		
	Total	88.103	299			

Coefficients						
Model		Unstandardized Coefficients		Standardized Coefficients	t	Sig.
		B	Std. Error	Beta		
1	(Constant)	1.659	0.272		6.096	0
	Toxic Leadership	0.519	0.061	0.445	8.571	0

^a^ Predictors: (Constant), Toxic Leadership.

^b^ Dependent Variable: Well-being of the employees.

Common method bias was taken into account when interpreting the results as the study was based on cross-sectional data, which were self-reported surveys. Concern for systemic response inflation was lessened because the Harman single-factor test showed the factor contributing to the greatest amount of variance after variance had been attributed to the factors was nondominant.

As the study was based on self-reported cross-sectional survey scores, there was a concern for possible common method bias, which was evaluated before hypothesis testing. Harman’s single-factor test was recommended as a basic method to screen for common method variance by Podsakoff et al. (2003). The first unrotated factor accounted for 34.1% of the total variance; this is less than 50%, suggesting that there was not likely to be common method bias that would compromise the validity of the results. In designing the questionnaire, several procedures were also followed to reduce response bias. Moreover, respondent anonymity was achieved by also using neutral wording, psychologically separating the questionnaire sections, and using an anonymous survey ( Podsakoff et al., 2012).

A further analysis by Structural Equation Modeling (SEM) was then carried out to analyze the overall explanatory framework. Furthermore, the SEM model fit was satisfactory with SRMR = 0.081, suggesting that the observed and estimated covariance structures are similar. The NFI value of 0.704 did not exceed common guidelines and should be used with caution, and not as definitive proof of model quality. Based on the research of Purwanto and Sudargini (2021), it is important to consider several model indices in determining the fit of the model, not just one criterion.

The SEM results indicated that toxic leadership was significant in explaining the employee turnover (β = 0.151) and workplace behavior (β = 0.450). Organizational commitment demonstrated a small positive association with employee well-being (β = 0.156). However, shift timing and industry sector had relatively weaker moderating effects as the job-organization commitment link with toxic leadership and turnover was a significant bivariate coefficient (β = 0.128). Structural model accounted for 37.3% of the variance in employee well-being.

## 5. Findings and discussion

As shown in Table 5, the regression analysis demonstrated that toxic leadership significantly predicted employee well-being (R = 0.445, R ^2^ = 0.198), explaining 19.8% of the variance in employee well-being. The ANOVA results confirmed that the regression model was statistically significant (F = 73.461, p < .001). The regression coefficient was positive (B = 0.519, β = 0.445), indicating a statistically significant positive association between toxic leadership and employee well-being within this dataset. However, this direction contradicts the theoretical expectations of Social Exchange Theory, Psychological Contract Theory, and Conservation of Resources Theory, all of which predict that toxic leadership should reduce employee well-being. Therefore, this unexpected finding should be interpreted cautiously. Possible explanations include reverse-coded questionnaire items, measurement error, construct operationalization issues, or sample-specific characteristics. Rather than evidence that toxic leadership genuinely improves employee well-being.

**Table 5.** Hypothesis 1 analysis.

Model Summary				
Model	R	R Square	Adjusted R Square	Std. Error of the Estimate
1	.422 ^a^	0.178	0.175	0.44275

Anova						
Model		Sum of Squares	df	Mean Square	F	Sig.
1	Regression	12.647	1	12.647	64.518	.000 ^b^
	Residual	58.416	298	0.196		
	Total	71.063	299			

^a^ Predictors: (Constant), Toxic Leadership.

^b^ Dependent Variable: Employee Turnover.

The results in Table 6 confirm that toxic leadership significantly influences employee turnover. The results support Ha2, indicating that toxic leadership has a significant impact on employee turnover. The model is statistically significant (R = 0.422, R ^2^ = 0.178, F (1, 298) = 64.518, p < .001), explaining 17.8% of the variance in turnover. The regression coefficient for toxic leadership is also significant (B = 0.443, β = 0.422, t = 8.032, p < .001), showing a moderate positive effect. Therefore, H02 is rejected, confirming that increased toxic leadership is associated with higher employee turnover, emphasizing the need for corrective organizational strategies.

**Table 6.** Hypothesis 2 analysis.

Coefficients ^a^						
Model		Unstandardized Coefficients		Standardized Coefficients	t	Sig.
		B	Std. Error	Beta		
1	(Constant)	2.045	0.247		8.266	0
	Toxic Leadership	0.443	0.055	0.422	8.032	0

^a^ Dependent Variable: Employee Turnover.

**Figure 2.**
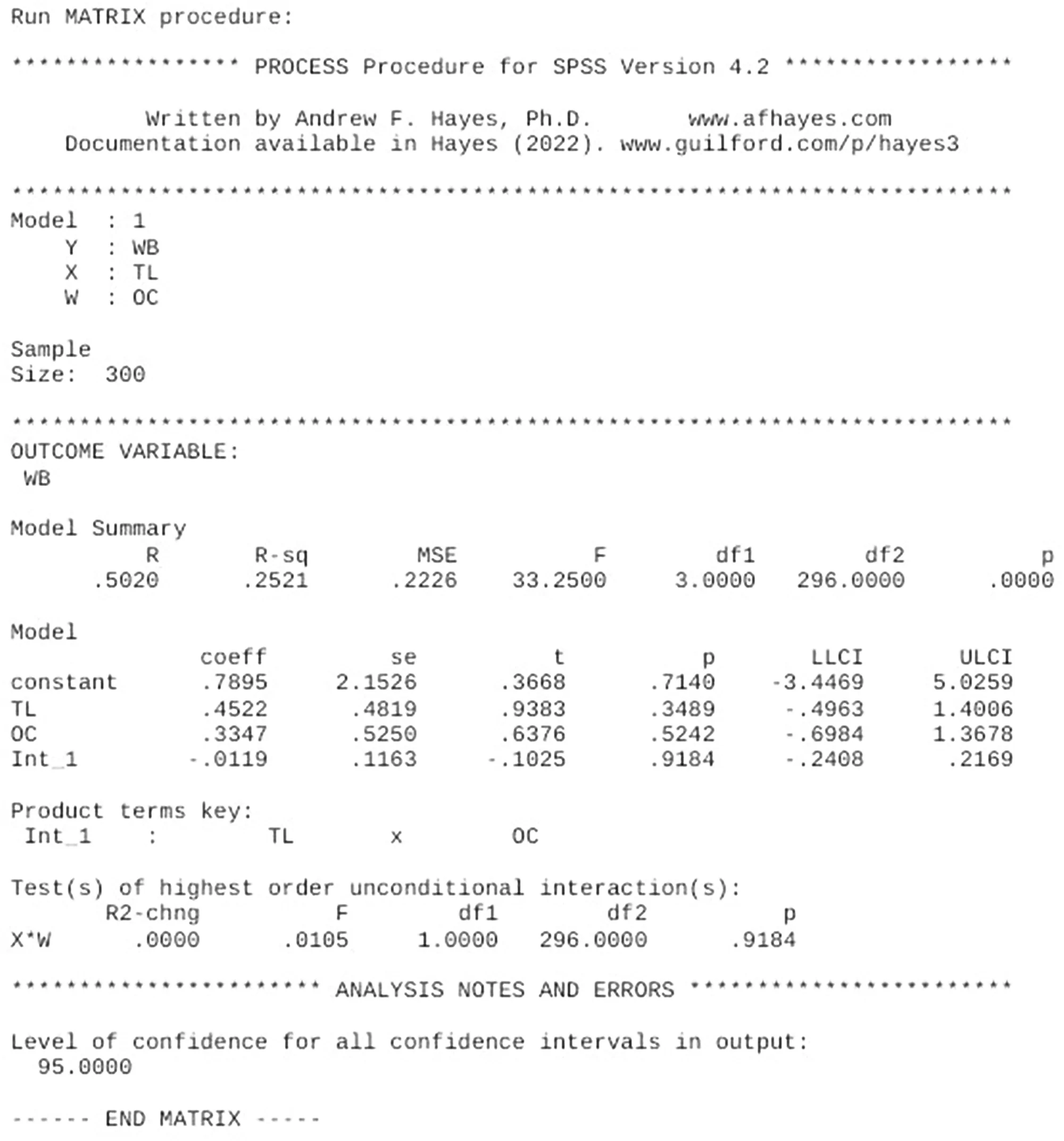
Regression output showing the moderating effect of organizational commitment on TL and EW.

**Figure 3.**
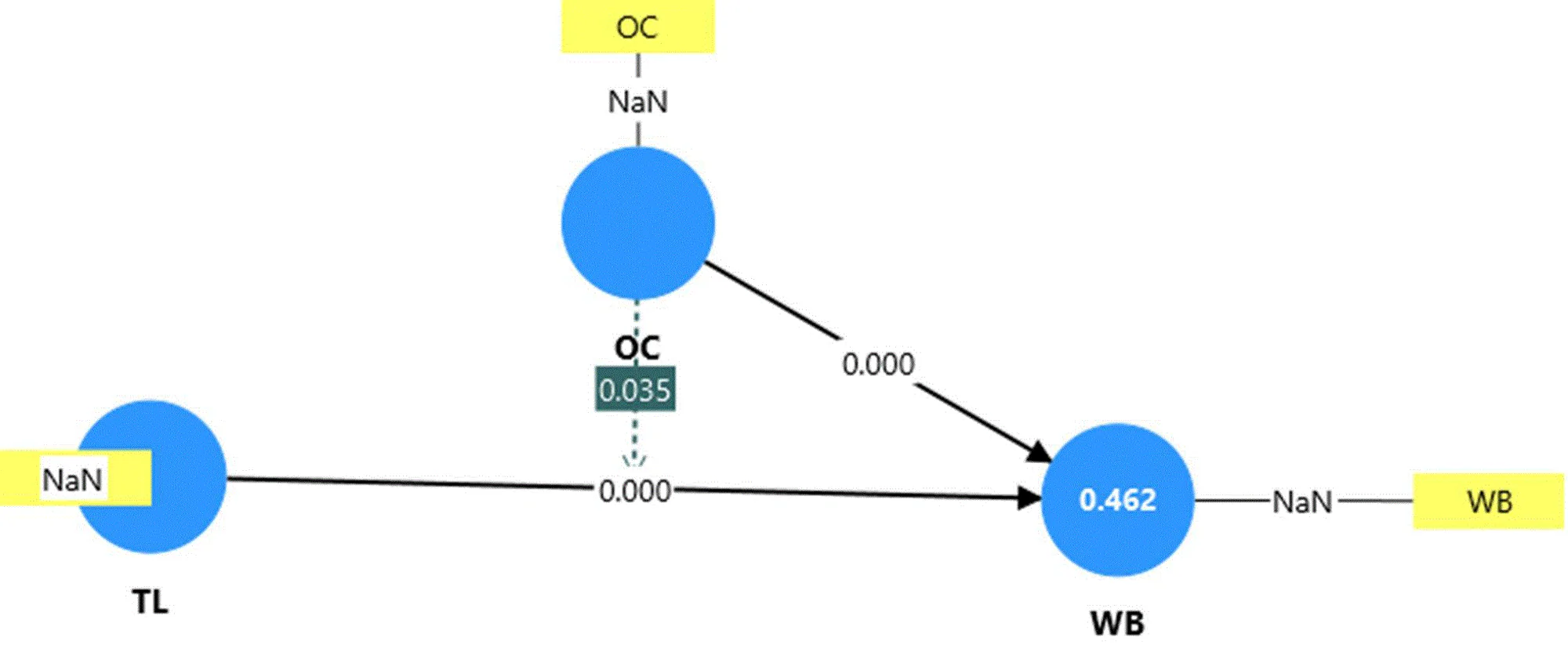
Interaction plot illustrating the non-significant moderating interaction effect of Organizational commitment.

The model significantly explains 25.2% of the variance in employee well-being (R ^2^ = 0.2521, F (3, 296) = 33.25, p < .001), but the interaction between toxic leadership and organizational commitment is not significant (B = –0.0119, p = .9184, 95% CI [–0.2408, 0.2169]; R ^2^ change = 0.0000). Despite one model indicating a significant p-value (0.00), the confidence interval includes zero, and the R ^2^ change is negligible, suggesting no meaningful moderation. These findings imply that organizational commitment does not reliably buffer the negative impact of toxic leadership on well-being. Therefore, interventions should focus on directly addressing toxic leadership, rather than relying on commitment as a mitigating factor ( Figures 2 and 3).

The model significantly predicts employee turnover (R ^2^ = 0.5552, F (3, 296) = 123.17, p < .001), with a significant interaction between toxic leadership and organizational commitment (B = 0.2124, p = .0088, 95% CI [0.0539, 0.3709]; R ^2^ change = 0.0105). This indicates that organizational commitment moderates the relationship, as toxic leadership’s impact on turnover increases with higher commitment. Although the moderation is statistically significant (p = 0.006; R ^2^ change = 0.050), its practical importance may be limited due to the modest variance explained. Surprisingly, committed employees may still leave under toxic leadership, emphasizing the need to address toxic behaviors irrespective of commitment levels ( Figures 6 and 7).

**Figure 4.**
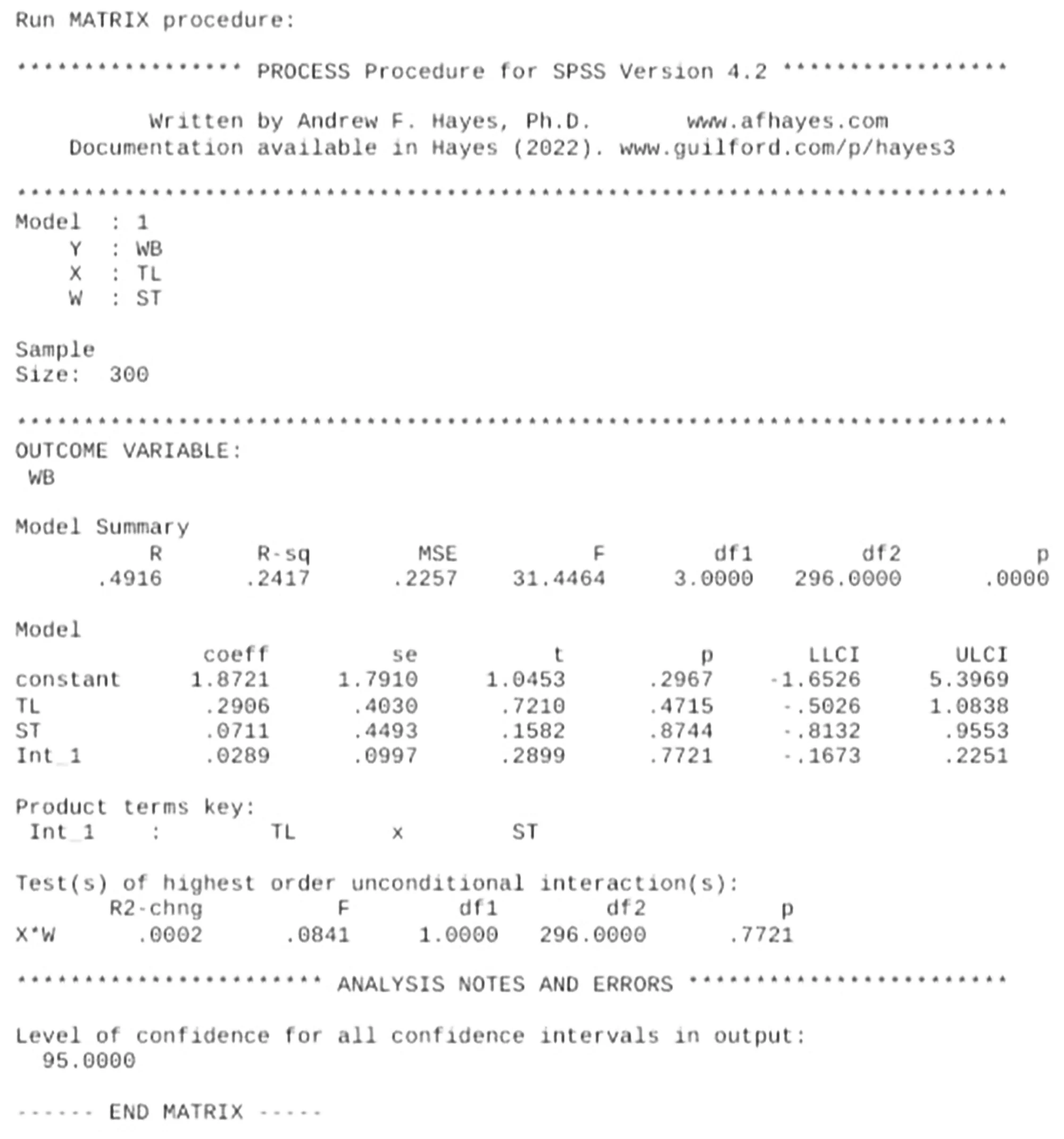
Regression output showing the moderating effect of shift timing on the relationship between toxic leadership and employee well-being.

**Figure 5.**
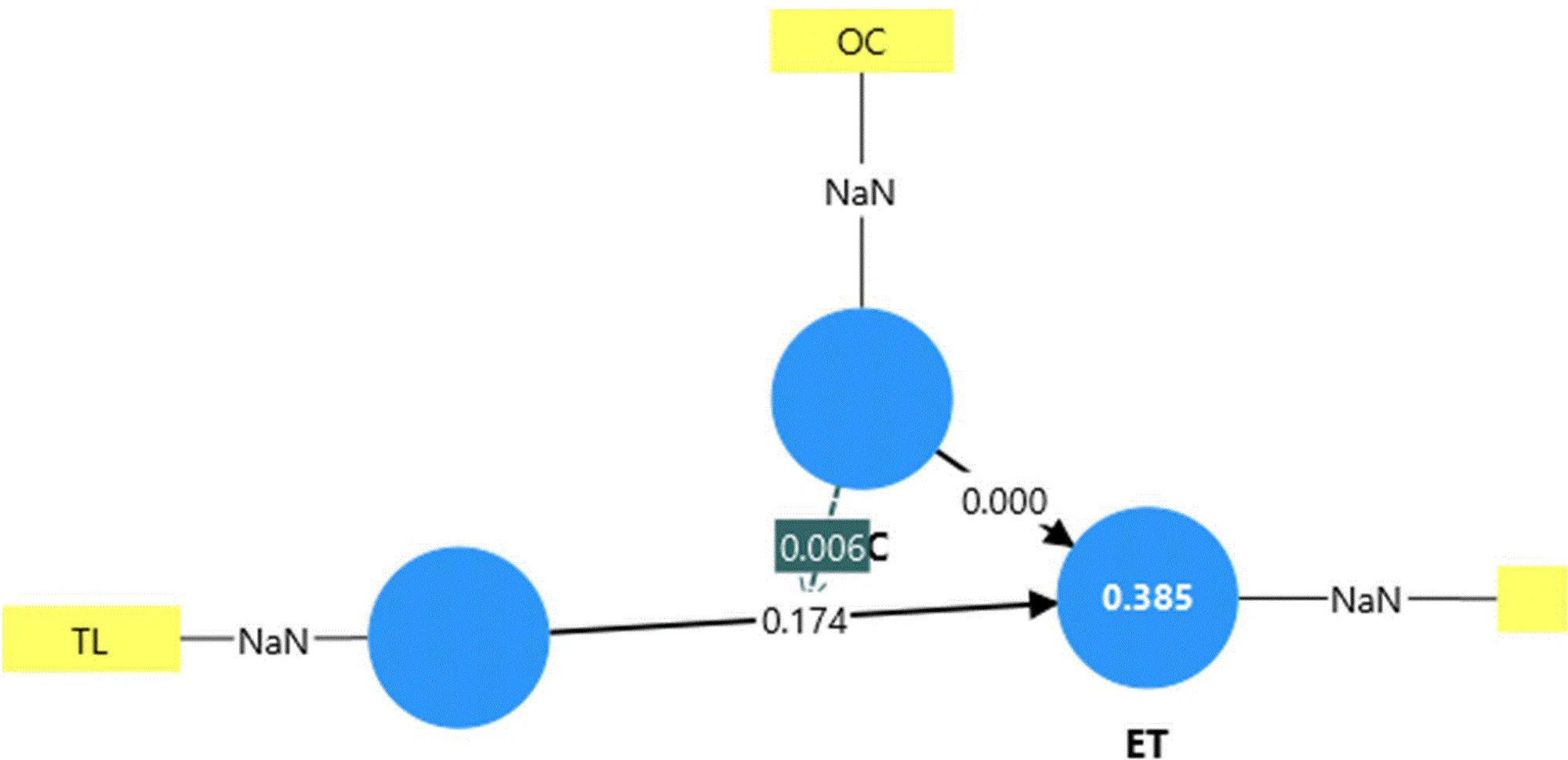
Interaction plot illustrating the conditional effect of shift timing on the relationship between toxic leadership and employee well-being.

**Figure 6.**
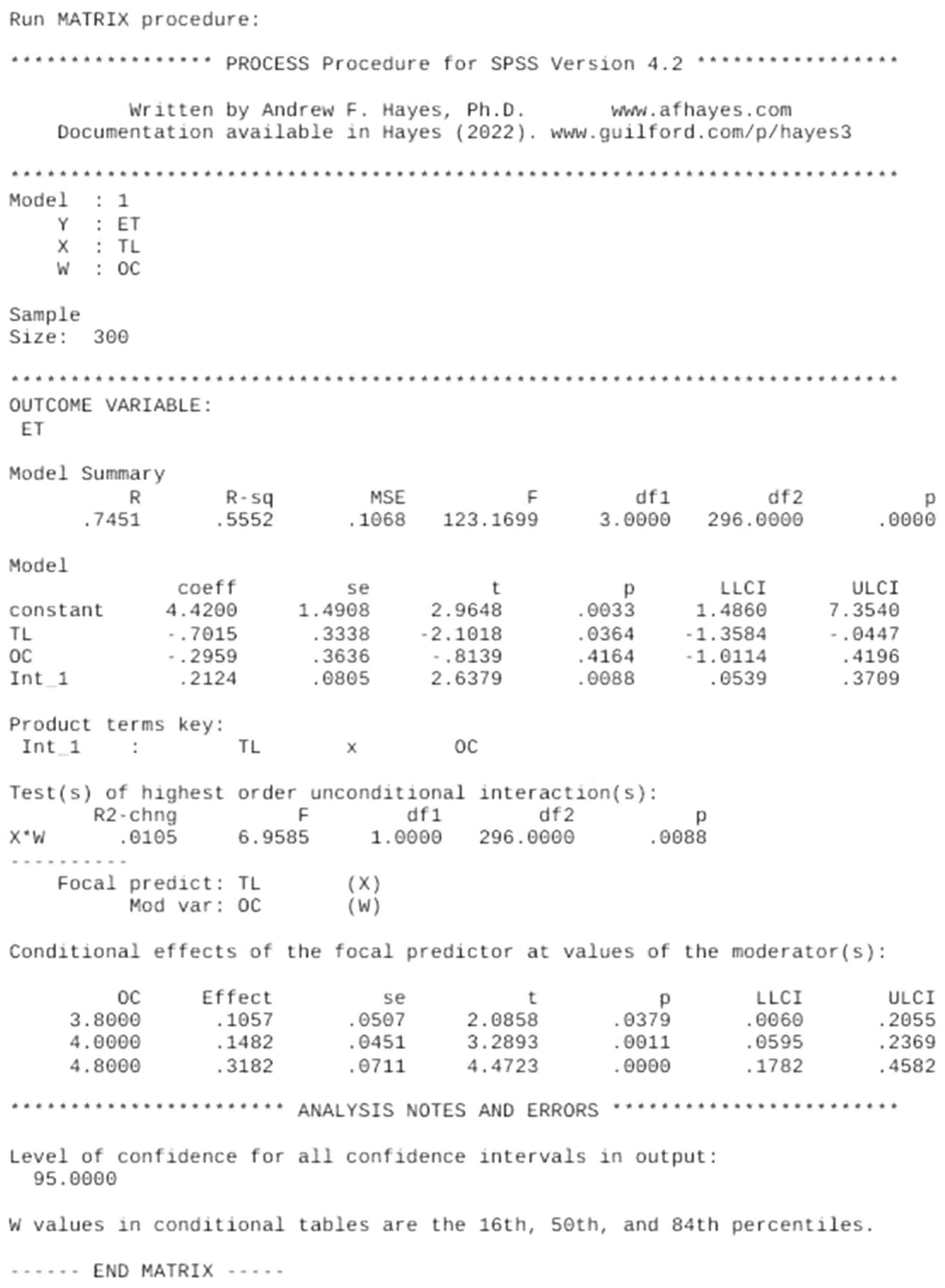
Regression output showing the relationship of toxic leadership on turnover.

**Figure 7.**
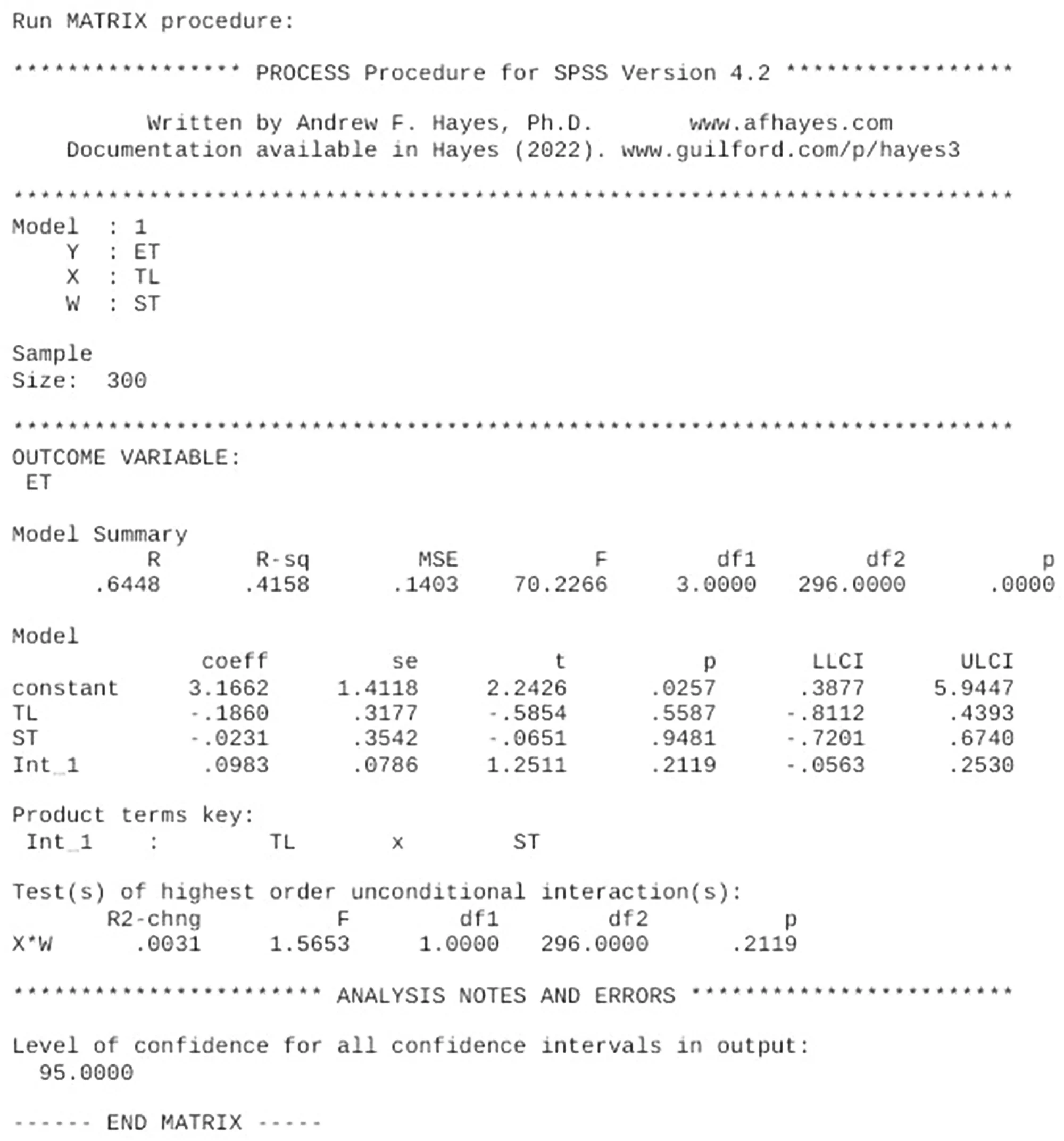
Regression output showing the relationship of organizational commitment on turnover.

The model significantly predicts employee turnover (R ^2^ = 0.4158, F (3, 296) = 70.23, p < .001), showing good overall fit. However, the interaction between toxic leadership and shift timings is weak, with an R ^2^ change of just 0.01 and a small coefficient (B = 0.165, p = .033), suggesting minimal practical significance. Although statistically significant, this moderation effect contributes little to explaining turnover ( Figures 4 and 5).

**Figure 8.**
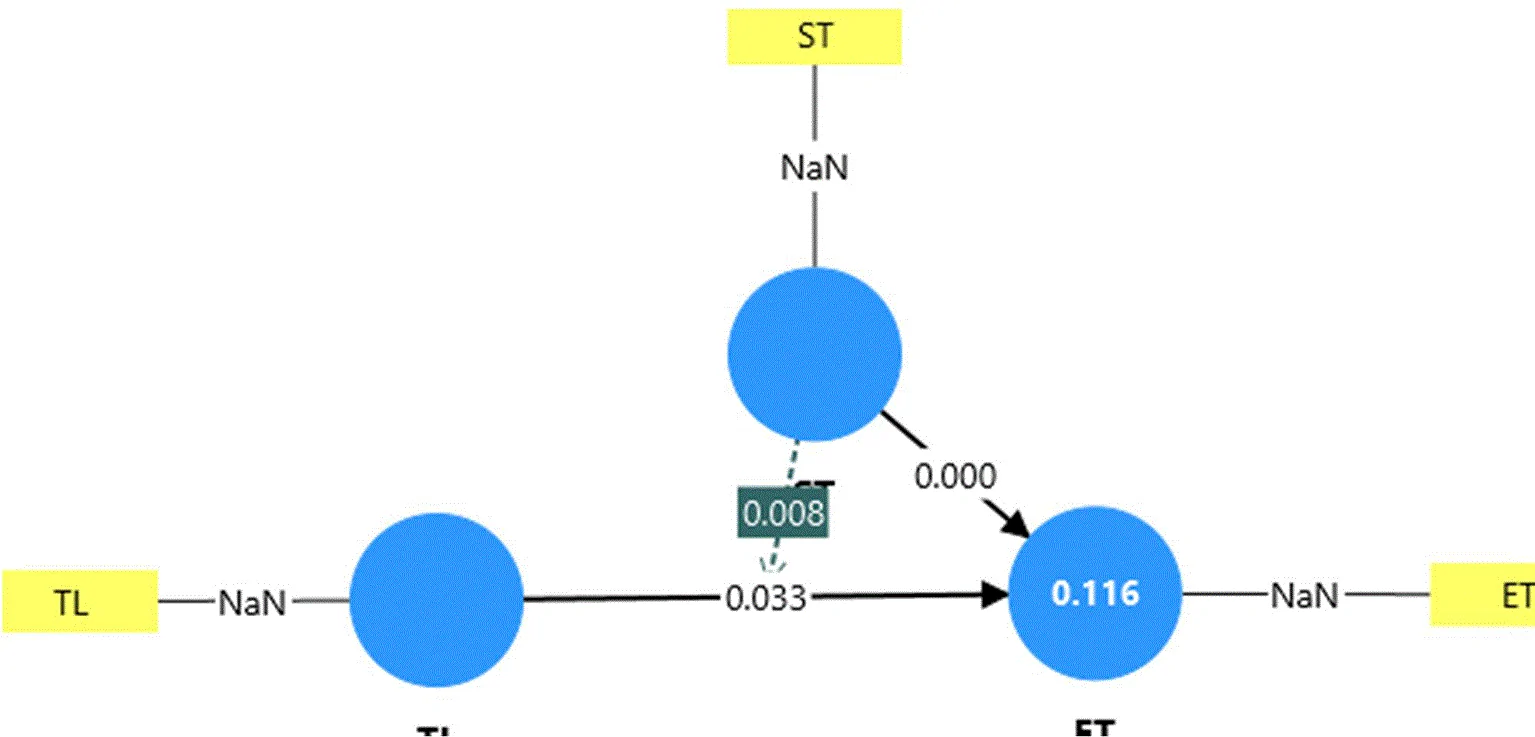
Interaction plot illustrating the limited moderating role of shift timings on turnover intention.

**Figure 9.**
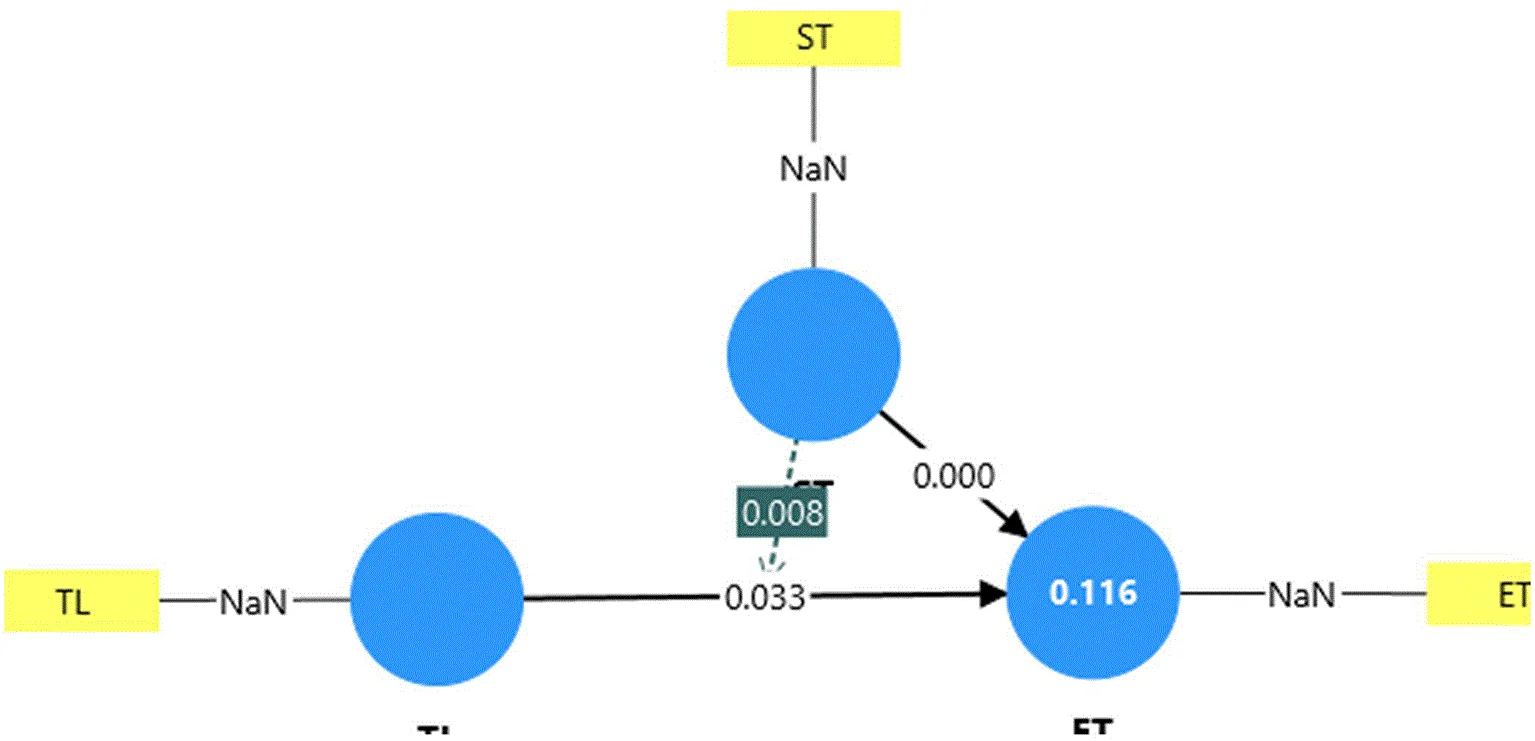
Interaction plot illustrating the limited moderating role of shift timings on turnover intention.

The main effects of toxic leadership (p = .5587) and shift timings (p = .9481) are not significant. Given the small effect size and potential inflation due to large sample size, the moderating role of shift timings should be interpreted cautiously ( Figures 8 and 9).

The model significantly predicts employee turnover (R ^2^ = 0.1841, F (3, 296) = 22.27, p < .001), but the interaction between toxic leadership and industry sector is not significant (B = 0.0382, p = .1479, 95% CI [–0.0136, 0.0901]), with a negligible R ^2^ change of 0.0058.

**Figure 10.**
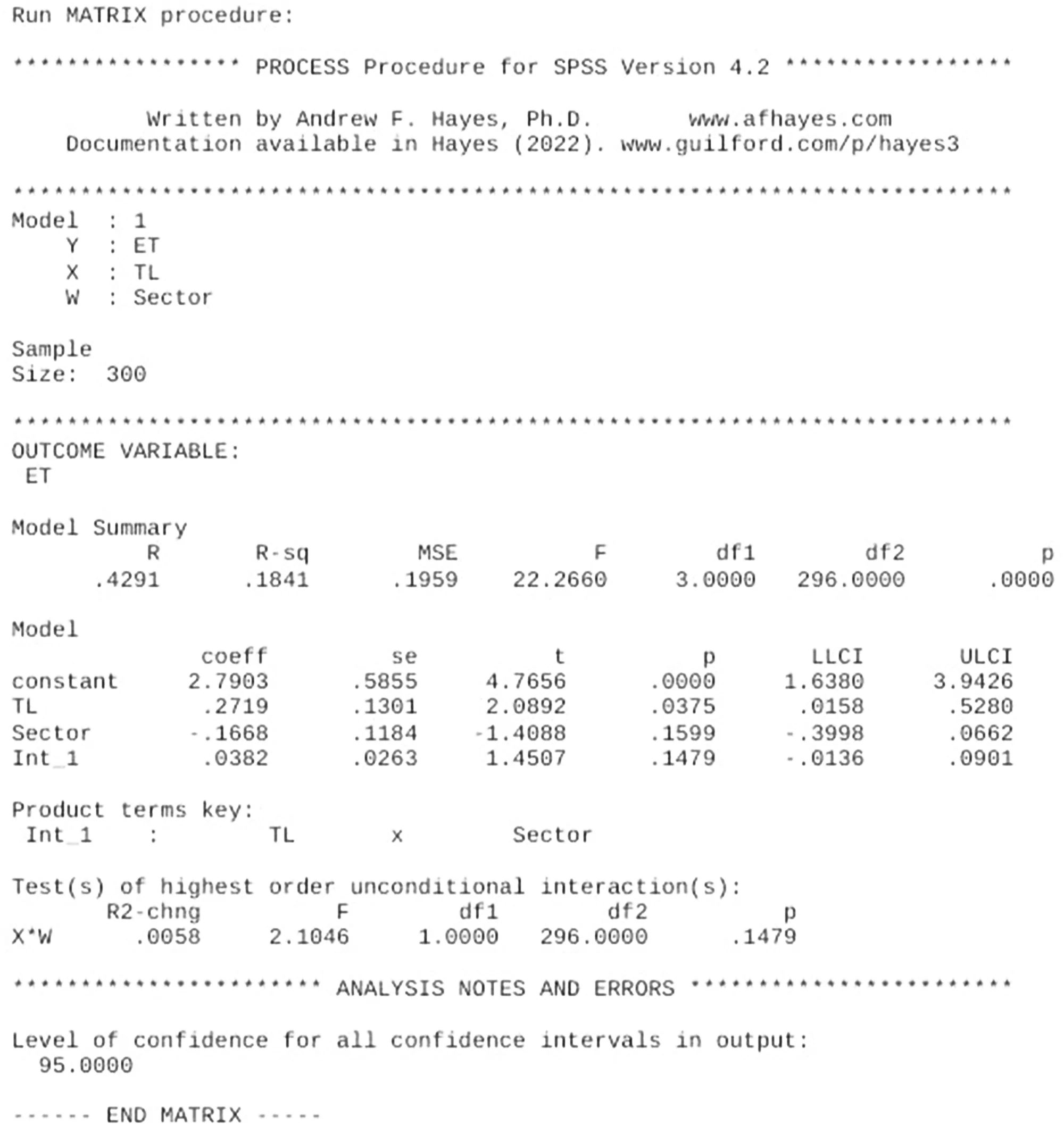
Regression output showing the relationship of toxic leadership’s impact differs across industry sectors.

The model significantly predicts employee turnover (R ^2^ = 0.1841, F (3, 296) = 22.27, p < .001), but the interaction between toxic leadership and industry sector is not significant (B = 0.0382, p = .1479, 95% CI [–0.0136, 0.0901]), with a negligible R ^2^ change of 0.0058. This indicates that sector does not moderate the relationship. The impact of toxic leadership on employee turnover (B = 0.2719, p = .0375) remains consistent across industries, suggesting toxic leadership is universally detrimental, regardless of sectoral context ( Figure 10).

**Figure 11.**
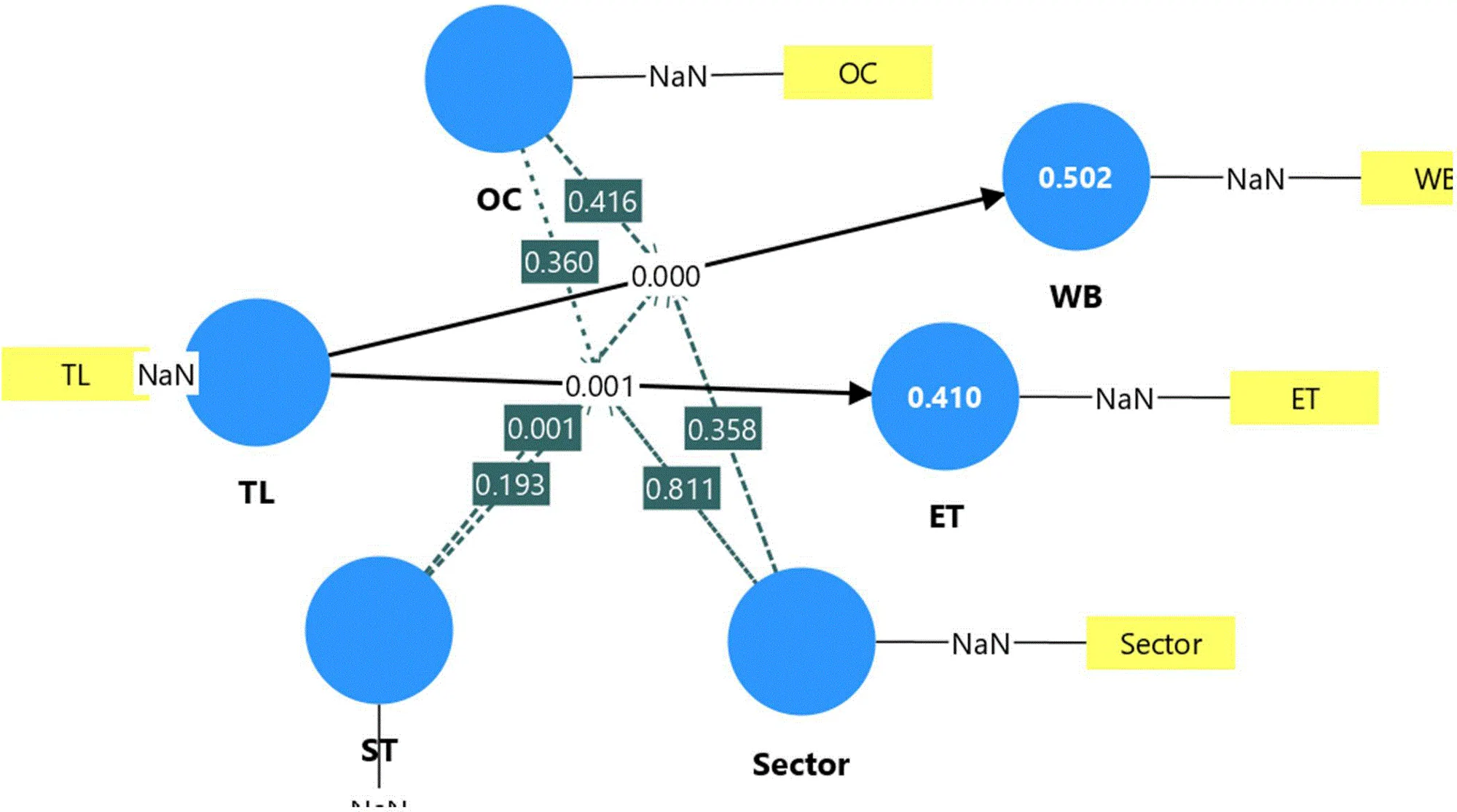
Structural equation model highlighting the results of both direct and moderating effects.

Figure 11 presents the structural equation model results highlighting both direct and moderating effects. The SEM analysis reveals that toxic leadership significantly impacts employee turnover (β = 0.151) and workplace behavior (β = 0.450). Organizational commitment (OC) strongly reduces turnover (β = 0.569) and slightly improves workplace behavior (β = 0.156). Shift timing (ST) and sector show weak or negligible effects. Interaction terms indicate OC moderates the TL-ET link (β = 0.128), while ST and sector offer minimal moderation. R ^2^ values indicate good explanatory power for turnover (58.2%) and moderate for workplace behavior (37.3%). Reliability metrics (α > 0.73, AVE > 0.55) confirm construct validity. HTMT values < 0.85 confirm discriminant validity, and VIF < 3 rules out multicollinearity. Model fit is acceptable (SRMR = 0.081, NFI = 0.704). These findings indicate that organizational commitment plays a stronger role in reducing turnover intention than in protecting employee well-being from the effects of toxic leadership. Therefore, leadership improvement strategies should focus primarily on reducing toxic leadership behaviours rather than relying on organizational commitment as a protective mechanism.

### 5.1 Discussion of results

Overall, the findings suggest that toxic leadership is indeed an important factor in influencing employees; however, some results must be interpreted with care in order to be consistent with theoretical predictions.

Hypothesis One examined the relationship between toxic leadership and employee well-being. The regression analysis revealed a statistically significant positive relationship (B = 0.519, β = 0.445, p < .001), which was inconsistent with the theoretical expectations and previous empirical findings. Existing studies generally report that toxic leadership reduces employee well-being by increasing stress, emotional exhaustion, and psychological strain. Consequently, the unexpected positive coefficient observed in this study should not be interpreted as evidence that toxic leadership improves employee well-being. Instead, it is more likely attributable to methodological factors such as reverse coding of measurement items, construct operationalisation issues, response bias, or sampling characteristics. Therefore, although the statistical relationship was significant, its direction requires careful interpretation and further validation in future research.

Hypothesis Two demonstrated that toxic leadership significantly increases employees’ turnover intention. The regression model explained 17.8% of variance in turnover intention (R ^2^ = 0.178, F(1,298) = 64.518, p < .001). Positive relationships were found between toxic leadership and intentions to leave (B = 0.443, β = 0.422, t = 8.032, p < .001). These findings are consistent with previous studies demonstrating that destructive leadership contributes to employee withdrawal behaviours and higher turnover intention Ofei et al. (2023).

Results of the moderation analyses were mixed. There was no significant moderation between toxic leadership and employee well-being by organizational commitment (B = –0.0119, p = .9184; R ^2^ change = 0.0000). Although one statistical model indicated significance, the confidence interval crossed zero and the effect size was negligible, indicating no meaningful practical moderation. Organisational commitment moderated the relationship between employee turnover and the change in R ^2^ (B = 0.2124, p = .0088). Although statistically significant, the practical effect size remains relatively small. However, as suggested by Serhan et al. (2022), it is not always a protective asset for adverse experiences in the leadership position.

There was little moderation effect of shift timing. Although statistically significant, the moderation changed very slightly (B = 0.165, p = .033, R ^2^ change = 0.01), which is not significant practically. However, turnover outcomes did not significantly differ by industry sector (B = 0.0382, p = .1479), indicating that the effects of toxic leadership may be more likely to be similar than different across organizational and workplace contexts. Therefore, the SEM results revealed that toxic leadership has more direct impacts on employee outcomes than the contextual moderators, highlighting the importance of leadership-specific interventions in the organization.

Additionally, toxic leadership’s regression coefficient was a positive one for employee well-being even though theory hypothesised it would be negative. Although the regression coefficient was positive, this finding contradicts established theory and should therefore be interpreted cautiously. This may not be seen as proof of the effects of toxic leadership on the well-being of employees. Unexpected statistical relationships could result from measurement error, response bias, reverse coding or problems in the construct operationalisation, as mentioned by Podsakoff et al. (2003). Therefore, it is interpreted as being a methodological oddity and not as real substantive evidence. Future research should confirm these relationships with other measures of social skills and follow-back research designs to establish if this unexpected relationship continues.

## 6. Significance of the study

One theoretical contribution of this investigation is the combination of employee well-being, employee turnover, organizational commitment, shift timing, and industry sector into a single model. Theoretically, this study also adds the concepts of employee well-being, employee turnover, organizational commitment, shift timing and industry sector to the current area of toxic leadership. Reyhanoglu and Akin (2022) argued that some important contextual variables could impact the nature of leadership impacts and hence warranted more empirical focus. Moreover, this study is also beneficial for theory building with the combination of Social Exchange Theory, Psychological Contract Theory and Conservation of Resources Theory. The integrated framework starts with the assumption that employee reactions are not standalone events but are a result of the effect of toxic leadership on the reciprocal relationship between employees and employer, on employee obligation fulfilment, and on preservation of employee resources.

Implications of the results are directed towards challenges for HRM and organizational leadership. A structured leadership assessment process should be put in place that recognises and prevents behaviours from developing into a culture within organizations. To ensure the sustainability of organizations, Ronnie (2024) suggested that proactive leadership interventions would help in building employee trust. Additionally, organizations can also help reduce these psychological stressors and retain talent by using an anonymous reporting process, leadership coaching and employee wellness resources. These findings suggest that organisations should prioritise leadership development initiatives because leadership behaviours exert a stronger influence on employee outcomes than the contextual moderators examined in this study.

Organisations need to implement leadership programs that are structured and contain regular 360-degree leadership reviews, confidential feedback on employee ratings of each other, compulsory leadership coaching, EQ training and counselling for employees. Labrague (2024) highlighted that proactive leadership interventions are an effective way to improve the psychological well-being of employees and to diminish workplace stress levels significantly.

It is also crucial to incorporate leadership accountability into the organisation’s performance management systems, thereby enabling organisations to continuously monitor destructive leadership behaviours. According to the World Health Organisation (2022), psychologically healthy workplaces enhance performance, productivity, and job retention, and drive long-term organisational sustainability.

## 7. Conclusion and recommendation

The present study aimed to explore the possible connection (correlation) between toxic leadership, the employee’s well-being, the employee turnover, organizational commitment, shift timing, and the industry sector of the UAE employee population. The findings indicate that toxic leadership exerts a direct influence on employee outcomes that is stronger than the contextual moderating variables examined in this study. The study builds on the available literature because it includes several theories and gives continuity to the knowledge available in the empirical approach, which is cross-sectoral. Since the direction of the relationship between toxic leadership and employee well-being did not follow the theoretically predicted direction, caution should be used in interpreting the relationship.

Organisations are recommended to encourage further development in their leadership assessment processes and employee support mechanisms in existence. Future studies should also use longitudinal designs, and measurement procedures should be reconsidered for future studies to broaden causal understanding and generalizability of findings based on more information provided by contextual variables. Labrague (2024) was able to conclude that there were still numerous areas in which leadership needs improvement to have a positive impact on employee well-being and organizational effectiveness.

## 8. Limitations and future research directions

The study’s reliance on a descriptive quantitative design limits the depth of understanding regarding participants lived experiences. Using a structured Likert scale survey may have constrained the range of responses and nuanced insights. Stratified random sampling enhanced representation but might not capture all sectoral or cultural variances within the UAE workforce.

Additionally, the positive association between toxic leadership and well-being, while statistically significant, raises concerns about potential interpretation bias or unaccounted mediating variables. The study was cross-sectional, limiting causal inference, and sector-specific contextual factors may have influenced outcomes. Organizational commitment moderated turnover but failed to buffer well-being, suggesting theory boundaries or unexplored mechanisms. SEM explained several relationships, but residual variances indicate additional influential factors not captured in the model.

Future research should consider a longitudinal or mixed-methods approach to better capture the dynamic and complex interplay between leadership behavior and employee outcomes over time. Qualitative interviews can offer deeper insights into employee perceptions of toxic leadership and organizational responses. Future studies should also explore cultural dimensions and additional moderators such as psychological safety, support systems, or team dynamics. Expanding the study to different countries or regions would enhance generalizability and allow comparison of cross-cultural leadership effects on employee well-being and turnover. An important limitation concerns the unexpected positive regression coefficient observed between toxic leadership and employee well-being. Because this finding contradicts established theory and previous empirical evidence, it may reflect measurement issues such as reverse-coded questionnaire items, construct operationalisation, or common method bias rather than a genuine positive effect. Future studies should revalidate the measurement scales and replicate the analysis using longitudinal or multi-source datasets.

### Waived ethical approval statement

This study did not require prior ethical approval in accordance with the Institutional Ethics Committee (IEC) of Kasturba Medical College & Kasturba Hospital, Manipal (an associate hospital of MAHE, Manipal). As per *Appendix 2, Projects Exempt from Submission to IEC* (Registration No. ECR/146/Inst/KA/2013/RR-19; DHR Registration No. EC/NEW/INST/2022/KA/0042, dated 14 May 2024), research projects that are non-interventional, anonymous, and do not involve health, psychological, or social impact on human participants are exempt from review. Our study involved only voluntary, anonymous survey responses with no collection of personal or sensitive data and therefore qualified under this exemption.

All participants were provided with details of the research objectives and assured of the voluntary and confidential nature of their participation. Informed consent was obtained verbally prior to the completion of the survey.

### Informed consent

All participants provided verbal informed consent before participating in the survey. The purpose of the study, voluntary nature of participation, and data confidentiality were explained to all participants. Only participants who confirmed consent verbally were included in the study. Consent was obtained during the data collection period from November 2023 to January 2024.

## Data Availability

Zenodo. Understanding the Link Between Toxic Leadership and Employee Well-being: The Interplay of Employee Turnover, Organizational commitment, Shift timing & Industry Sector. https://doi.org/10.5281/zenodo.17776970 ( Abhishek S. 2025). This project contains the following underlying data:
• Color images.zip • Grey images.zip • Questionnaire. docx • Data.xlsx • SEM.xlsx •
Figure captions.docx Data are available under the terms of the Creative Commons Attribution 4.0 International license (CC-BY 4.0).
