# Supplementary material for: Understanding the Link Between Toxic Leadership and Employee Well-being: The Interplay of Employee Turnover, Organizational commitment, Shift timing & Industry Sector
Source: F1000Res. 2026 Aug 8;15:35. Originally published 2026 Jan 9. [Version 2] doi: 10.12688/f1000research.171577.2 (PMC13560036; doi:10.12688/f1000research.171577.2)
Supplement: Peer review history

# Reviewer report: Reviewer response for version 2

**DOI:** 10.5256/f1000research.206329.r509057

**Published:** 2026-09-03

**Authors:** Aarti Sharma

**Affiliations:** 1 IIHMR UNIVERSITY, Jaipur, Rajasthan, India

Approved

Is the work clearly and accurately presented and does it cite the current literature?

Yes

If applicable, is the statistical analysis and its interpretation appropriate?

Yes

Are all the source data underlying the results available to ensure full reproducibility?

No source data required

Is the study design appropriate and is the work technically sound?

Yes

Are the conclusions drawn adequately supported by the results?

Yes

Are sufficient details of methods and analysis provided to allow replication by others?

Yes

Reviewer Expertise:

NA

I confirm that I have read this submission and believe that I have an appropriate level of expertise to confirm that it is of an acceptable scientific standard.

# Reviewer report: Reviewer response for version 2

**DOI:** 10.5256/f1000research.206329.r509058

**Published:** 2026-09-03

**Authors:** Khushboo Kumar

**Affiliations:** 1 Department of Management & Commerce, School of Management Sciences Varanasi, Varanasi, Uttar Pradesh, India

1. The manuscript needs a stronger research gap, supported by citations. Hypotheses should be supported by relevant prior empirical studies rather than unsupported general statements.

 2. Only SRMR and NFI are reported although five fit indices are claimed.

 3. Use of PLS with AMOS is contradictory.

 4. Discussion must explicitly connect findings with the research gap and objectives. Justification of unsupported hypotheses is also required.

 5. The manuscript needs to be better structured and systematically organized to ensure a clear and logical flow of arguments, improve coherence across sections and facilitate readers’ understanding of the study.

Is the work clearly and accurately presented and does it cite the current literature?

Partly

If applicable, is the statistical analysis and its interpretation appropriate?

Partly

Are all the source data underlying the results available to ensure full reproducibility?

No source data required

Is the study design appropriate and is the work technically sound?

Partly

Are the conclusions drawn adequately supported by the results?

Partly

Are sufficient details of methods and analysis provided to allow replication by others?

Partly

Reviewer Expertise:

Human Resource Management, Organizational Behaviour

I confirm that I have read this submission and believe that I have an appropriate level of expertise to confirm that it is of an acceptable scientific standard, however I have significant reservations, as outlined above.

# Reviewer report: Reviewer response for version 2

**DOI:** 10.5256/f1000research.206329.r513148

**Published:** 2026-08-28

**Authors:** Lista Meria

**Affiliations:** 1 Esa Unggul University, West Jakarta, Indonesia

1. By convention, quantitative research articles typically structure the Introduction section according to the following sequence: research urgency, followed by a brief literature review, then the research gap or novelty, followed by the research objectives, and concluding with the research contribution. In this article, the research gap is not presented in the Introduction section; rather, it is elaborated only in a separate subsection following the subsection on Research Objectives and Targets. As a result, the research objectives are presented without being preceded by a conceptual justification regarding the gap the study intends to address, so that the argumentative flow becomes less coherent for the reader.

 2.The theoretical framework subsection should first be elaborated narratively without reference to the hypothesis codes (H1 through H7), for example with a sentence such as "this theory underlies the hypothesis regarding the direct relationship between toxic leadership and employee well-being, which will be elaborated in the following subsection." The formal hypothesis codes should be introduced for the first time only in the Hypothesis Development subsection, rather than being mentioned earlier in the theoretical framework subsection.

 3.The Hypothesis Development subsection (3.7) opens with the statement that all seven hypotheses were developed "with reference to Social Exchange Theory, Psychological Contract Theory, and Conservation of Resources Theory." However, upon examining the justification paragraph for each hypothesis, only one of the seven hypotheses (H2) is explicitly supported by a citation of prior empirical research, namely the reference to Gandolfi and Stone (2022). The remaining six hypotheses (H1, H3, H4, H5, H6, and H7) are formulated solely through general narrative statements without a single reference to prior empirical studies; for example, the sentence "Previous studies have reported inconsistent findings regarding whether highly committed employees are better able to withstand negative leadership experiences," used to justify H3, is not followed by any citation of the "previous studies" referred to.

 This condition is fundamentally at odds with the positivist research paradigm the authors claim to have adopted in this study (see Section 4, which states that the research "adopts a positivist research paradigm, which emphasizes objectivity, measurement, and hypothesis testing"). Within the positivist tradition, hypotheses should be derived deductively from established theory and reinforced by an accumulation of prior empirical evidence, so that the argumentative trail from general theory to specific prediction can be traced and verified by the reader. When five of the seven hypotheses are formulated without reference to any supporting empirical study, a gap emerges between the methodological claim (a rigorous positivist approach) and the actual writing practice (hypothesis justifications consisting of assertions or general statements without a traceable empirical basis).

 4.The conceptual framework essentially constitutes a visual representation of the research hypotheses that have been formulated. However, each path representing the relationship between variables in the conceptual framework figure is not labeled with its corresponding hypothesis code, requiring readers to refer back to the text to understand which hypothesis each path represents.

 5.The statement "the overall methodology of the research process is outlined in Table 1" does not correspond to the actual content of the table. Table 1 is not a flow chart of the research methodology, but rather a communalities table resulting from factor analysis. This discrepancy is further confirmed by the title of Table 1 itself ("RM Flow Chart"), which likewise does not accurately represent the table's content.

 6.The questionnaire items presented are not grouped or labeled according to the variable/construct being measured, such as toxic leadership, employee well-being, or turnover intention. Without this grouping, readers find it difficult to verify the correspondence between each item and the construct it is intended to represent.

 7.A number of the figures presented in the article are inadequate, and several appear to be cropped. This condition makes it difficult for readers to independently interpret the content of the figures without repeatedly referring back to the main text.

 8.The article does not present a summary table consolidating the hypothesis testing results for all hypotheses (H1 through H7). For example, a table with columns for the hypothesis code, coefficient, p-value, and decision (accepted/rejected). The absence of such a table makes it difficult for readers to obtain a comprehensive overview of the testing results without having to trace through all the results paragraphs separately.

 9.Section "4. Research Methodology," particularly subsection 4.1 Statistical Analysis, should by convention describe only the analytical procedure employed, that is, the type of statistical test, software, and evaluation criteria applied. However, this subsection instead already includes actual results from the analysis, including the SRMR value (0.081), NFI (0.704), β coefficients from the SEM model, the results of Harman's single-factor test (34.1%), as well as the reliability and Average Variance Extracted (AVE) values for each construct.

 As a result, the research results are divided between two separate sections: some are presented in Section 4 (labeled Methodology) and others in Section 5 (labeled Findings and Discussion, which contains Tables 5, 6, and so on). This inconsistent separation makes it difficult for readers and reviewers to distinguish what constitutes research procedure from what constitutes actual findings, and represents a fairly significant structural violation of the IMRaD (Introduction, Methods, Results, and Discussion) convention, which requires a clear boundary between the Methods section ("what was done") and the Results section ("what was found").

 10. The Methodology section states that model fit evaluation was conducted using five indices, namely SRMR, NFI, CFI, TLI, and RMSEA. However, in the Results section, actual values are reported for only two of these indices, namely SRMR (0.081) and NFI (0.704); the actual values for CFI, TLI, and RMSEA are never mentioned anywhere in the manuscript.

 11. The manuscript states that SPSS AMOS software was used together with a Partial Least Squares (PLS) estimation procedure. This statement contains a significant technical error, because AMOS is software for Covariance-Based SEM (CB-SEM), which uses Maximum Likelihood (ML) estimation rather than PLS. PLS-SEM procedures are typically run using software such as SmartPLS or WarpPLS, not AMOS.

 If the authors indeed applied PLS-SEM, then the software name stated is incorrect; conversely, if AMOS was indeed used, then the estimation method is ML/CB-SEM rather than PLS. This discrepancy needs to be clarified, given that the two approaches involve fundamentally different assumptions, criteria for interpreting model fit indices, and formats for reporting results.

 12. The Discussion section does not explicitly link the research findings back to the research gap and research objectives presented in the Introduction. Ideally, the Discussion should affirm the extent to which the research findings succeed in addressing the previously identified gap, so that the research contribution is conveyed coherently rather than standing apart from the argumentative framework established at the beginning of the article. This is especially important for hypotheses that were not supported (failure to reject H0), which require in-depth justification from the authors.

 13. The Abstract states that one of the principal strengths of this study is its cross-sector analysis across various industries in the UAE. However, the article does not explicitly present a comparative analysis across sectors. For example, a comparison of the pattern of relationships among variables in the healthcare sector versus manufacturing, retail, and other sectors.

 Recommendation:

 1. Move the subsection "Research Objectives and Targets" to the concluding part of the Introduction that is, after the research gap has been explained or reverse the order of the two subsections. In this way, the research gap precedes the formulation of the objectives, and the argumentative flow becomes more logical and easier for the reader to follow.

 2. The Theoretical Framework and Hypothesis Development subsections should be merged into a single subsection, in which each theoretical discussion is immediately followed by the corresponding H0/Ha statement in sequence, namely, Social Exchange Theory for H1 and H2, Psychological Contract Theory for H3 and H4, and Conservation of Resources Theory for H5, H6, and H7, so that no gap remains between the introduction of a hypothesis code and its substantive definition.

 3. Strengthen each hypothesis justification paragraph, particularly for H1, H3, H4, H5, H6, and H7 with citations of relevant prior empirical research, so that each hypothesis is genuinely derived from an accumulation of existing evidence and theory, consistent with the claimed positivist paradigm, rather than resting on an untraceable narrative assertion.

 4. Include hypothesis code labels (H1, H2, and so on) on each path in the conceptual framework figure so that readers can directly map each path to the hypothesis it tests.

 5. Revise the reference sentence in the text as well as the title of Table 1 to be consistent with the table's actual content, for example by changing it to "Communalities Table of Measurement Items."

 6. Group the questionnaire items according to the variable/construct being measured, thereby facilitating the assessment of the instrument's content validity by both readers and reviewers.

 7. Provide each figure with an informative caption and ensure that all figures are displayed in full, without cropping, prior to resubmission of the article.

 8. Add a single summary table of hypothesis testing results that consolidates the hypothesis code, coefficient, p-value, and decision status.

 9. Move all actual statistical values (model fit indices, coefficients, reliability/validity test results) currently located in Section 4.1 to the Results section, so that the Methodology section contains only procedures and evaluation criteria, without any findings figures.

 10. Clarify and correct the actual combination of software and estimation method used, so that it is consistent with the analytical procedure reported.

 11. Add a concluding paragraph to the Discussion section that explicitly links each key finding to the research gap and objectives stated in the Introduction, together with adequate justification for hypotheses not supported by the data.

 12. Present additional analysis to support this cross-sector claim (for example, through difference tests or multi-group analysis across sectors); if such analysis was not conducted, the claim of cross-sector strength in the Abstract should be revised so as not to mislead readers.

Is the work clearly and accurately presented and does it cite the current literature?

Partly

If applicable, is the statistical analysis and its interpretation appropriate?

Partly

Are all the source data underlying the results available to ensure full reproducibility?

Partly

Is the study design appropriate and is the work technically sound?

Partly

Are the conclusions drawn adequately supported by the results?

Partly

Are sufficient details of methods and analysis provided to allow replication by others?

Partly

Reviewer Expertise:

Human Resources Management, Organizational Behavior, Management Science, Information Systems

I confirm that I have read this submission and believe that I have an appropriate level of expertise to confirm that it is of an acceptable scientific standard, however I have significant reservations, as outlined above.

# Reviewer report: Reviewer response for version 1

**DOI:** 10.5256/f1000research.189198.r494900

**Published:** 2026-07-08

**Authors:** Aarti Sharma

**Affiliations:** 1 IIHMR UNIVERSITY, Jaipur, Rajasthan, India

**1. Theoretical Framing and Conceptual Clarity**

 The tripartite theoretical framework—Social Exchange Theory, Psychological Contract Theory, and Conservation of Resources Theory—is well-integrated and appropriately applied. Each theory addresses a distinct mechanism, and their collective explanatory power is convincing. A brief synthesized paragraph mapping each theory to its corresponding hypotheses would enhance clarity. I would also encourage the authors to consider integrating the emerging construct of *moral injury*, which would provide a compelling explanatory mechanism for the unexpected finding that organizational commitment amplifies turnover intentions under toxic leadership—a paradox that existing frameworks only partially illuminate.

**2. Methodology and Research Design**

 The cross-sectional design is appropriate for the research aims, and the stratified random sampling approach enhances external validity. The sample of 300 respondents across diverse UAE industries provides adequate statistical power. Psychometric properties are robust: Cronbach's α > 0.73, AVE > 0.55, HTMT < 0.85, and VIF well within acceptable limits. The verbal consent protocol is ethically justified given cultural sensitivities around workplace anonymity in the UAE; a brief elaboration on these contextual considerations would strengthen the ethical narrative for international readers.

**3. Analysis and Interpretation of Results**

 The statistical analysis is technically proficient and interpreted with commendable caution. The authors correctly distinguish between statistical and practical significance—too often neglected in this field. While the organizational commitment moderation achieves significance ( *p* = .008), the modest R² change (0.0105) is appropriately flagged. The anomalous positive coefficient for toxic leadership predicting well-being is handled with scientific integrity: transparently acknowledged as a likely measurement artifact with discussion of potential suppression effects. The SEM fit indices (χ²/df = 2.36, CFI = 0.91, SRMR = 0.081) indicate satisfactory fit. Supplementing *p*-values with effect sizes (e.g., Cohen's *f*²) would further enhance interpretive precision.

**4. Originality and Contribution**

 The study makes a meaningful contribution through its simultaneous examination of multiple moderators in a non-Western context—a combination that distinguishes it from prior work. The unexpected finding that organizational commitment may amplify turnover intentions represents a genuine theoretical contribution, challenging conventional assumptions about commitment as a protective resource. The UAE contextual focus adds valuable geographic diversity to a Western-dominated literature.

**5. Writing Quality and Organization**

 The manuscript is well-organized with logical flow. Minor refinements would enhance readability: streamlining occasional repetitions between results and discussion, ensuring consistent terminology, and improving resolution of SEM path diagrams. The discussion appropriately emphasizes interpretation over statistical restatement.

**Practical Implications**

 The central recommendation—that organizations prioritize direct intervention against toxic leadership rather than relying on employee commitment as a buffer—is both evidence-based and practically urgent. The finding that committed employees may be at heightened turnover risk has important implications for retention strategies. Expanding discussion on operationalizing anti-toxic leadership interventions would strengthen the practical contribution.

Is the work clearly and accurately presented and does it cite the current literature?

Yes

If applicable, is the statistical analysis and its interpretation appropriate?

Yes

Are all the source data underlying the results available to ensure full reproducibility?

No source data required

Is the study design appropriate and is the work technically sound?

Yes

Are the conclusions drawn adequately supported by the results?

Yes

Are sufficient details of methods and analysis provided to allow replication by others?

Yes

Reviewer Expertise:

Emotional Intelligence, work engagement, Employee well-being, and recruitment.

I confirm that I have read this submission and believe that I have an appropriate level of expertise to confirm that it is of an acceptable scientific standard.

# Reviewer report: Reviewer response for version 1

**DOI:** 10.5256/f1000research.189198.r473513

**Published:** 2026-05-21

**Authors:** Khushboo Kumar

**Affiliations:** 1 Department of Management & Commerce, School of Management Sciences Varanasi, Varanasi, Uttar Pradesh, India

**Recommendation: Major Revision**

 This manuscript examines the relationship between toxic leadership, employee well-being, and turnover intention, while also exploring the roles of organizational commitment, shift timing, and industry sector. The topic is timely and relevant, particularly given increasing organizational concerns regarding employee mental health, retention, and workplace culture.

 The paper demonstrates effort in integrating theoretical perspectives and applying quantitative analysis techniques, including regression analysis and SEM. The practical relevance of the study is evident, and the inclusion of contextual variables such as shift timing and sector adds some value to the discussion. However, the manuscript currently has several conceptual, methodological, and interpretative issues that should be addressed before the paper can be considered for publication. They are as follows:

**1. Theoretical Framing and Conceptual Clarity**

 The manuscript draws upon Social Exchange Theory, Psychological Contract Theory, and Conservation of Resources Theory to explain the impact of toxic leadership. While these theories are relevant, their integration remains somewhat descriptive. The paper would benefit from a clearer explanation of how these theories complement one another and how each specifically supports the proposed hypotheses.

 In addition, some constructs require clearer conceptual distinction. Terms such as employee well-being, workplace behavior, organizational commitment, and turnover intention are discussed throughout the paper, but their relationships are not always clearly differentiated.

 A major concern relates to the finding that toxic leadership shows a positive relationship with employee well-being. This result appears inconsistent with the theoretical foundation and with prior literature cited in the manuscript. Although the discussion acknowledges a possible measurement issue, this concern requires much deeper examination. The authors should carefully review:

- reverse coding procedures,
- questionnaire scaling,
- construct operationalization,
- possible statistical suppression effects.

At present, this issue significantly weakens confidence in the interpretation of the findings.

**2. Methodology and Research Design**

 The quantitative approach and use of regression analysis and SEM are generally appropriate for the study objectives. However, several methodological details require clarification.

 The manuscript should provide more information regarding:

- sampling procedures,
- respondent demographics,
- sector representation,
- response rates,
- inclusion criteria.

Although stratified random sampling is mentioned later in the paper, the methodology section should explain this process more systematically.

 The factor analysis results indicate strong item loadings across constructs. However, the validation procedures remain incomplete for an SEM-based study. The paper should present a clearer measurement model assessment, including:

- Cronbach’s alpha,
- Composite Reliability,
- AVE,
- discriminant validity measures,
- and HTMT ratios.

Additionally, since all data were collected through self-reported surveys at a single point in time, the study should address the possibility of common method bias.

 The section on ethical approval also requires clarification. Although the study was classified as exempt, the manuscript investigates potentially sensitive workplace experiences. A clearer explanation of ethical safeguards and participant protection procedures would strengthen the study’s credibility.

**3. Analysis and Interpretation of Results**

 The statistical analysis is reasonably detailed, and the inclusion of moderation effects adds analytical depth. However, some interpretations require greater caution and consistency.

 For example, the manuscript repeatedly states that toxic leadership negatively affects well-being, while the regression coefficient reported is positive. This contradiction needs to be resolved clearly.

 The moderation analyses also require more balanced interpretation. In several cases, statistically significant interactions appear to have very small practical effects due to negligible changes in explained variance. While the manuscript occasionally acknowledges this, the discussion later tends to overstate the implications.

 The SEM section would also benefit from clearer reporting. The paper should specify:

- the SEM software used,
- model estimation procedures,
- model fit justification,
- the rationale for combining regression analysis with SEM.

The reported NFI value appears relatively low and should be discussed more critically.

**4. Originality and Contribution**

 The topic is relevant and practically important. However, toxic leadership and turnover intention have already received substantial scholarly attention. The manuscript would therefore benefit from a stronger explanation of:

- the specific research gap with citations
- the novelty of integrating shift timing and sector
- the study’s unique contribution to existing literature.

At present, many findings appear to confirm existing studies rather than extend theory in a meaningful way.

**5. Writing Quality and Organization**

 The manuscript would benefit considerably from language editing and structural refinement.

 Some sections contain:

- repetitive explanations,
- duplicated statements,
- inconsistent terminology,
- grammatical issues.

For example, parts of the SEM findings are repeated almost verbatim in the discussion section. Streamlining the presentation would improve readability and overall coherence.

 The discussion section should also focus more on interpreting the findings rather than restating statistical outputs.

**Practical Implications**

 The study offers useful practical insights for HR professionals and organizational leaders. The emphasis on leadership development, employee support systems, and ethical workplace practices is relevant and timely.

 However, the paper could strengthen its practical contribution by discussing:

- how organizations can operationalize anti-toxic leadership interventions,
- the economic costs associated with toxic leadership and turnover,
- and the broader implications for employee mental health and workplace sustainability.

Is the work clearly and accurately presented and does it cite the current literature?

Partly

If applicable, is the statistical analysis and its interpretation appropriate?

Partly

Are all the source data underlying the results available to ensure full reproducibility?

No source data required

Is the study design appropriate and is the work technically sound?

Partly

Are the conclusions drawn adequately supported by the results?

Partly

Are sufficient details of methods and analysis provided to allow replication by others?

Partly

Reviewer Expertise:

Human Resource Management, Organizational Behaviour

I confirm that I have read this submission and believe that I have an appropriate level of expertise to confirm that it is of an acceptable scientific standard, however I have significant reservations, as outlined above.

# Response

**Published:** 2026-07-23

**Authors:** Abhishek Shukla

**Affiliations:** School of Business, Manipal Academy of Higher Education - Dubai Campus, Academic City, Dubai, United Arab Emirates

We sincerely thank the reviewer for the valuable comments and constructive suggestions. The manuscript has been carefully revised, and all changes have been highlighted in the attached tracked-changes version.

**Comment 1. Theoretical Framing and Conceptual Clarity**

**Response:**

 Thank you for this valuable observation. The theoretical framing of the manuscript has been strengthened to provide a clearer explanation of the complementary roles of Social Exchange Theory (SET), Psychological Contract Theory (PCT), and Conservation of Resources (COR) Theory in explaining the effects of toxic leadership on employee outcomes. The revised manuscript now explains more clearly how SET accounts for reciprocal employee responses to leadership behaviour, PCT explains employees' reactions when perceived organisational obligations are not fulfilled, and COR Theory explains the depletion of employees' emotional and psychological resources under toxic leadership.

 Collectively, these theoretical perspectives provide a stronger foundation for the proposed hypotheses and improve the overall conceptual coherence of the study. Conceptual clarity has also been improved throughout the manuscript by consistently distinguishing between employee well-being, organisational commitment, turnover intention, and workplace behaviour, with terminology standardised across all sections. References to "workplace behaviour" within the structural model have been revised to ensure consistency with the study constructs. The concern regarding the unexpected positive relationship between toxic leadership and employee well-being has also been addressed.

 The revised manuscript now explicitly acknowledges that this finding is inconsistent with the theoretical expectations and previous empirical literature. The discussion has been expanded to examine possible methodological explanations, including reverse coding procedures, questionnaire scaling, construct operationalisation, response bias, and potential statistical suppression effects, and the finding is interpreted more cautiously rather than being presented as evidence that toxic leadership improves employee well-being.

**Comment 2. Methodology and Research Design**

**Response:**

 The methodology section has been revised to provide greater clarity and transparency regarding the research design. The sampling strategy has been described more systematically by expanding the explanation of the stratified random sampling procedure, respondent selection process, sector representation, response rate, and the inclusion and exclusion criteria. Additional demographic information has also been incorporated to provide a clearer profile of the study sample. To strengthen the measurement model assessment, the revised manuscript now reports Cronbach's alpha, Composite Reliability (CR), Average Variance Extracted (AVE), discriminant validity, the Fornell–Larcker criterion, HTMT ratios, and Variance Inflation Factor (VIF), thereby strengthening the assessment of construct reliability and validity. The possibility of common method bias has also been addressed through both procedural and statistical approaches.

 The manuscript now describes measures adopted during questionnaire administration to minimise response bias and reports the results of Harman's Single Factor Test to assess the potential influence of common method variance. The ethical considerations have likewise been expanded. Although the study was exempt from formal ethical review, the revised manuscript now provides a clearer explanation of the procedures implemented to safeguard participants, including informed consent, voluntary participation, confidentiality, anonymity, secure data handling, and participants' right to withdraw from the study.

**Comment 3. Analysis and Interpretation of Results**

**Response:**

 The analysis and interpretation of the findings have been revised to improve consistency and provide a more balanced discussion. The manuscript now acknowledges that the positive regression coefficient observed between toxic leadership and employee well-being is inconsistent with the theoretical framework and previous empirical evidence. Rather than drawing definitive conclusions from this unexpected result, the discussion has been expanded to consider possible methodological explanations, including measurement-related issues, and the finding is interpreted with appropriate caution.

 The interpretation of the moderation analyses has also been refined by distinguishing between statistical significance and practical significance. Where interaction effects produce only limited increases in explained variance, the discussion now recognises that their practical implications should be interpreted cautiously.  The Structural Equation Modelling (SEM) section has also been strengthened by clearly identifying the software used (IBM SPSS Amos), describing the model estimation procedures, reporting the model fit indices, and explaining the rationale for combining regression analysis with SEM. In addition, the discussion now provides a more balanced interpretation of the model fit statistics, including the reported NFI value, by evaluating the overall model fit using multiple established indices rather than relying on a single measure.

**Comment 4. Originality and Contribution**

**Response:**

 The research gap section has been revised to provide a more coherent explanation of the rationale for the study and the limitations of previous research. The manuscript now emphasises the integrated examination of toxic leadership, employee well-being, turnover intention, organisational commitment, shift timing, and industry sector within a single conceptual framework, while highlighting the relevance of examining these relationships within the multicultural organisational context of the United Arab Emirates. The discussion of the study's contribution has also been strengthened by providing a clearer explanation of its theoretical, methodological, and practical significance. Rather than examining these variables in isolation, the study brings them together within a unified framework, thereby offering additional insights into the mechanisms through which toxic leadership influences employee outcomes across diverse organisational settings.

**Comment 5. Writing Quality and Organization**

**Response:**

 The manuscript has undergone language editing and structural revision to improve clarity, readability, and overall coherence. Repetitive explanations and duplicated material have been reduced, terminology has been standardised, and grammatical issues have been corrected to improve the overall quality of the manuscript. The discussion section has also been revised to place greater emphasis on interpreting the findings in relation to the theoretical framework and existing literature while reducing unnecessary repetition of statistical results already presented in the Results section. These revisions have improved the organisation, consistency, and overall readability of the manuscript. This version stays closely aligned with the revisions reflected in the manuscript, avoids overstating the extent of the changes, and reads in a natural academic style appropriate for an author response to reviewers.
